# Neuropilin-2 Expression Promotes TGF-β1-Mediated Epithelial to Mesenchymal Transition in Colorectal Cancer Cells

**DOI:** 10.1371/journal.pone.0020444

**Published:** 2011-07-01

**Authors:** Camille Grandclement, Jean René Pallandre, Séverine Valmary Degano, Erika Viel, Adeline Bouard, Jérémy Balland, Jean-Paul Rémy-Martin, Benoit Simon, Alain Rouleau, Wilfrid Boireau, Michael Klagsbrun, Christophe Ferrand, Christophe Borg

**Affiliations:** 1 INSERM UMR 645, Besançon, France; 2 University of Franche-Comté, Besançon, France; 3 EFS Bourgogne Franche-Comté, Besançon, France; 4 Department of Pathology, CHU Besançon, Besançon, France; 5 Department of Medical Oncology, CHU Besançon, Besançon, France; 6 FEMTO-ST Institute, University of Franche Comté, Besançon, France; 7 Department of Surgery and Pathology, Children's Hospital, Boston, Massachusetts, United States of America; Emory University, United States of America

## Abstract

Neuropilins, initially characterized as neuronal receptors, act as co-receptors for cancer related growth factors and were recently involved in several signaling pathways leading to cytoskeletal organization, angiogenesis and cancer progression. Then, we sought to investigate the ability of neuropilin-2 to orchestrate epithelial-mesenchymal transition in colorectal cancer cells. Using specific siRNA to target neuropilin-2 expression, or gene transfer, we first observed that neuropilin-2 expression endows HT29 and Colo320 for xenograft formation. Moreover, neuropilin-2 conferred a fibroblastic-like shape to cancer cells, suggesting an involvement of neuropilin-2 in epithelial-mesenchymal transition. Indeed, the presence of neuropilin-2 in colorectal carcinoma cell lines was correlated with loss of epithelial markers such as cytokeratin-20 and E-cadherin and with acquisition of mesenchymal molecules such as vimentin. Furthermore, we showed by surface plasmon resonance experiments that neuropilin-2 is a receptor for transforming-growth factor-β1. The expression of neuropilin-2 on colon cancer cell lines was indeed shown to promote transforming-growth factor-β1 signaling, leading to a constitutive phosphorylation of the Smad2/3 complex. Treatment with specific TGFβ-type1 receptor kinase inhibitors restored E-cadherin levels and inhibited in part neuropilin-2-induced vimentin expression, suggesting that neuropilin-2 cooperates with TGFβ-type1 receptor to promote epithelial-mesenchymal transition in colorectal cancer cells. Our results suggest a direct role of NRP2 in epithelial-mesenchymal transition and highlight a cross-talk between neuropilin-2 and TGF-β1 signaling to promote cancer progression. These results suggest that neuropilin-2 fulfills all the criteria of a therapeutic target to disrupt multiple oncogenic functions in solid tumors.

## Introduction

Neuropilins (NRPs) are transmembrane non-tyrosine kinase glycoproteins originally described in the nervous system. Neuropilin (NRP) family consists of two genes, neuropilin-1 (NRP1) and neuropilin-2 (NRP2). During nervous system development, NRP1 and NRP2 play a critical role in axon retraction and guidance by binding class III semaphorins [1]. Initially characterized as neuronal receptors, NRPs were also found to be expressed in endothelial cells and subsequently were shown to play a role in the development of the vascular system [2].

NRPs display a short intracytoplasmic tail which does not contain a kinase domain. Initial investigations of neuropilin-dependent molecular pathways suggested that neuropilins can not directly transmit intracellular signals. This led to the proposal that hetero-dimerization with other receptors are required to mediate neuropilin-downstream signaling. One of these co-receptor complexes described so far involves vascular endothelial growth factor receptor (VEGFR) [3], [4], [5]. Besides the amplification of VEGFR signaling, NRPs might interact with plexins to mediate class 3 semaphorin signal transduction via Rho-related G proteins, modulating cytoskeleton organization [6].

Nevertheless, a highly conserved amino-acid sequence promoting NRPs intracellular tail binding to the PDZ domain of GAIP-C terminus interacting protein-1 (GIPC-1) was recently reported suggesting the possibility that NRPs might regulate alternative biological functions [7].

The multiple functions of NRPs were recently highlighted by the identification of NRP role in oncogenesis. Besides the presence of NRPs on tumor-associated vessels, NRPs were expressed by a large variety of tumors, suggesting a potential role of this glycoprotein in cancer progression. Indeed, NRP2 expression was found in osteosarcoma [8], melanoma [9], lung cancers [10], [11], brain tumors [12], [13] colon cancers [14], pancreatic cancers [15], [16], [17], breast cancers [18], myeloid leukemia [19], salivary adenoid cystic carcinoma [20], infantile hemangioma [21], ovarian neoplasms [22] and bladder cancers [23]. In colon carcinoma, NRP2 directly promotes tumor progression in a cell autonomous manner (see review of NRP2 expression on cancer cells in Table S1). It was suggested that NRP2 oncogenic properties rely on an increased VEGFR1 phosphorylation and an activation of the VEGFR1/Pi3K/Akt signaling. [14] However, the precise molecular pathways driven by NRP2 and involved in oncogenesis remain largely unknown.

Epithelial-mesenchymal transition (EMT) is one of the major molecular mechanisms carried out during oncogenesis to promote cancer progression. EMT is characterized by a breakdown of cell junctions, the loss of epithelial characteristics and cell polarity, contributing to carcinoma progression. Besides the gain of mesenchymal markers, EMT endows cancer cell for migration, invasiveness and subsequent metastasis formation [24]. Despites several studies pertaining to the role of NRP2 in cancer progression, no substantial evidence established an involvement of this molecular pathway in EMT.

Here, we used colon cancer cell lines transfected with NRP2 transgene or siRNA to investigate NRP2 involvement in EMT. These experiments provided evidence that NRP2 endows colon cancer cell lines for colony and xenograft formation. Moreover, a conversion from epithelial to fibroblast-like shape was triggered by NRP2 expression, as well as the acquisition of vimentin and EMT specific transcription factors. Then, we examined the influence of NRP2 on transforming growth factor β1 (TGF-β1) signaling that is believed to contribute to the late-stage carcinoma by inducing EMT. We showed that NRP2 promotes a constitutive Smad2/3 phosphorylation in colon cancer cell lines. Moreover, specific siRNA targeting NRP2 or treatment with pharmacological inhibitors of TGFβ-1 type 1 receptor (TGFβRI) prevented Smad2/3 phosphorylation and the NRP2-mediated EMT of colorectal cancer cells. Collectively, these results suggest that NRP2 cooperates with TGFβRI to promote EMT in colorectal carcinoma.

## Materials and Methods

### Cell culture

Human cell lines HT29, Colo320, SW620, MCF7, Caki, A498, HEK293 were purchased from the American Type Cell Culture Collection and were cultured in RPMI1640 or DMEM (Lonza, Paris, France) supplemented with 10% heat inactivated endotoxin free fetal calf serum (FCS), (Invitrogen, Cergy-Pontoise, France). Bes-PAC01, Bes-PAC03, Bes-PAC04 and Bes-PAC05 (Pancreatic Adeno-Carcinoma) cell lines were originally isolated from ascitic fluids derived from four patients with pancreatic adenocarcinomas, in our university hospital. R3III cell line was kindly provided by Nathalie Labarriere, Inserm (Nantes, France). Jijoye and Raji cell lines (Human Burkitt Lymphoma) were provided by Diaclone (Besançon, France). Cell lines used in this study were authentified using DNA profiling (short tandem repeats analysis), in line with ATCC's recommendations (see Table 1). Short tandem repeat (STR) analysis is a molecular biology method recommended for cell line identification. Cell lines used in this study were genotyped before freezing and every two months. STR analysis was performed with the AmpFISTR Identifiler PCR Amplification Kit (Applied Biosystems). Height specific loci including tandem repeats on DNA from cancer cell lines were analyzed. STR analysis measures the exact number of repeating units for each allele (D7S820 8,20 means that 8 and 20 repeats are identified on each allele of the D7S820 locus for the cell line Jijoye). If a variant appears that contains a partial repeat, that partial repeat unit is designated by a decimal followed by the number of bases in the partial repeat.

**Table 1:** DNA STR (Short Tandem Repeats) profiling of tumor cell lines.

	D7S820	CSF1PO	TH01	D13S317	D16S539	vWA	TPOX	D5S818
Jijoye	8,10	10,11	7,9	12	10,11	15,19	6,8	12
SW620	8,9	13,14	8	12	9,13	16	11	13
Caki-1	8,12	10,11	6,8	11,12	12	15,17	8,11	11,12
MCF-7	8,9	10	6	11	11,12	14,15	9,12	11,12
A498	10,11	11,12	6, 9.3	12	12	18	8,11	11,13
Colo320	9,12	11	8,9	11	11,12	15,18	8,9	12
HT29	10	11,12	6,9	11,12	11,12	17,19	8,9	11,12
5637	10,11	11	7,9	11	9	16,18	8,9	11,12
HEK293	11,12	11,12	7,9.3	12,14	9,13	16,19	11	8,9
MDAMB231	8,9	12,13	7,9.4	13	12	15,18	8,9	12
Raji	10	10,12	6,7	13	8,11	16,19	8,13	10,13

### pcDNA Expression plasmids

The influence of NRP2 on colon cancer cell progression was assessed by transferring NRP2 gene in the HT29 cell line. We generated HT29^NRP2^ cell lines using two expression plasmids encoding hNRP2 (pcDNA3.1-NRP2, kindly provided by M. Klagsbrun) and pCMV6-XL5-NRP2 (purchased from Origene (Rockville, MD, USA). Control HT29 cells were generated using pcDNA3.1 or pCMV6-XL5 vectors. 1.5×10^6^ HT29 cells were seeded in a 60 mm^3^ flask in 4 mL medium and incubated for 24 h. Then, cells were stably transfected with 1 µg of pcDNA3.1-NRP2 or pCMV6-XL5-NRP2 expression vectors or control vectors using the Effectene kit (Qiagen, Courtaboeuf, France) according to the manufacturer's instruction. Cells transfected with pcDNA3.1 vectors were selected with 0.8 mg/mL of geneticin (Invitrogen, Cergy-Pontoise, France), 48 h following the transfection.

### Small Interfering RNA

Using the Ambion siRNA web design tool, we identified one potential NRP2-specific target sequence. Specific NRP2 siRNA (sense 5′-AAA GGC TGG AAG TCA GCA CTA ATT T-3′ and anti-sense 5′-AAA AAT TAG TGC TGA CTT CCA GCT T-3′) and scramble siRNA (sense 5′-AAAGGAGGGGCATGCCACGTTGG-3′ and anti-sense 5′-AAAACCAACGTGGCATGCCCCTC-3′) sequences were annealed and cloned into the BbsI site of the 3′ LTR of pFIV-H1/U6 vector according to manufacturer's instructions (System Biosciences, Mountain View, CA). Sequences were confirmed by NIH BLAST analysis to have no substantial homology to sequences in other vertebrate genes. Lentiviral supernatant production and subsequent infection of cells were performed according to manufacturer's instructions (System Biosciences, Mountain View, CA). Colo320 stably transfected were selected with 3 µg/mL of puromycin (Invitrogen, Cergy-Pontoise, France), 48 h following the transfection.

### Flow cytometry

Anti-NRP2, anti-NRP1, anti-pSmad2/3, anti-TGFβ1, anti-TGFβR1 were from Santa-Cruz Biotechnology (Heidelberg, Germany). Anti-smad2/3 and anti-TGFRII, were from RD system (Lille, France). Alexafluor488-labelled secondary antibodies were purchased from Fluoprobes (Interchim, Montluçon, France). Ten thousands cells from each sample were evaluated for fluorescence detection using BD FACSCanto cytometer. (Becton Dickinson, Le Pont de Claix, France) For intracellular staining, cells were fixed for 20 min at 4°C in 2% paraformaldehyde. Staining was then realized at room temperature for 30 min in a buffer containing 0.4% saponin and 5% FCS. Wash buffer contained 0.1% saponin, 5%FCS. For flow cytometry analysis, Relative Fluorescence Intensity (RFI) was calculated.

### Cell Proliferation Assay

Cell proliferation in vitro was analyzed with the tetrazolium salt 3-(4,5-dimethylthiazol-2-yl)-2,5 diphenyltetrazolium bromide (MTT). Briefly, 4000 cells per well were seeded in 96-well micro-plates containing 100 µL of medium per well. For analysis, 10 µL of MTT substrate (of a 5 mg/mL stock solution in phosphate-buffered saline) was added to each well, and the plates were let to standard tissue incubator conditions for an additional 2 hours. Cells were solubilized in 200 µL of dimethyl sulfoxide, and colorimetric analyses were performed (wavelength, 570 nm). The plates were assayed every 24 hours for next 3 consecutive days.

### ELISA assay

Cells were incubated in RPMI or DMEM-1% FCS for 24 h. Then cells were counted and 10000 cells per well were seeded in 96-microplate in 200 µL DMEM-0.1% FCS for 24 h. VEGF production was assessed on supernatants using human VEGF Elisa kit (Strathmann Biotec, Hamburg, Germany).

### Flow Cytometry Analysis of DNA Content

Cells were harvested by trypsinization, washed with ice-cold PBS, fixed in 70% ethanol. Prior to DNA analysis, cells were stained with 50 µg/mL propidium iodide (Sigma-Aldrich, Lyon, France) and 2 µg/mL DNase-free RNase (Sigma-Aldrich, Lyon, France) for 15 min at 37°C in the dark. DNA content was measured using a FACSCalibur flow cytometer (Becton Dickinson, Le Pont de Claix, France) and analyzed with the CellQuest program.

### Xenograft experiments

Nude mice were obtained from Janvier (Le Genest St Isle, France), and maintained in our animal facility according to the Animal Experimental Ethics Committee Guidelines. 1×10^6^ cells of HT29^ctrl^, HT29^NRP2^, Colo320^si-RNA-NRP2^ and Colo320^siRNA-ctrl^ cell lines resuspended in 100 µL of PBS were inoculated subcutaneously in nude mice and tumor growth was monitored biweekly in each group. Tumor volume was calculated by the formula *V* = 1/2 *a* × *b* ^2^, where *a* is the longest tumor axis, and *b* is the shortest tumor axis. When tumors reached 1 cm in diameter, mice were sacrificed and tumors were fixed in formol for subsequent immunohistochemical analysis.

### Colony formation assay

Effect of NRP2 expression on colony formation in vitro was evaluated by soft agar colony-formation assay. 5000 cells per well were seeded in 500 µL of 2% agarose medium in a 24-well plate. Cells were incubated at 37°C, 5% CO2 and photos were taken after 10 days of culture.

### Invasion assay

Invasion was evaluated using 96W QCM Invasion Assay (Millipore, USA). Briefly, equal number (100000) of control cells (HT29^ctrl^) or NRP2 expressing cells (HT29^NRP2^) resuspended in serum-free medium were placed in the top compartment of a standard 8 µM pore Boyden chamber. Feeder tray wells contained serum free medium or medium 10% FCS. Following 16 hours invasion (37°C, 5% CO2), invasive cells were incubated with cell detachment buffer, lysed and marked with CyQuant GR dye. (QCM 96W Assay, Millipore, International). Fluorescence was then evaluated with a fluorescence plate reader (Cell Lab Quanta, Beckman-Coulter) using a 480–520 nm filter set.

### Cell Treatments

TGF-β1 signaling was inhibited using SD-208 or SB-431542 (TGFRI kinase inhibitor) diluted in DMSO (Sigma-Aldrich, Lyon, France). DMSO served as control medium in all experiments. In some experiments Avastin (Pharmacy of CHU Jean Minjoz, Besançon, France) was used to inhibit VEGF-A. TGF-β1 was purchased from RD System (Lille, France).

### Western Blot analysis

Briefly, after two washing steps, cells were harvested and solubilized in lysis buffer containing1 mM EDTA, 1 mM NaF, 1 mM Vanadate and one complete Mini protease Inhibitor Cocktail Tablet (Complete Mini EDTA Free, per 10 mL of lysis buffer, Roche, France). 30 µg of whole-cell lysates were separated by sodium duodecyl sulfate-polyacrylamide gel electrophoresis and transferred to polyvinylidene difluoride membranes by electroblotting. The blots were then blocked for 1 h in 5% milk before incubation with specific antibodies as follows: anti-NRP2 and anti-twist1 (Santa Cruz Biotechnology, Heidelberg, Germany), anti-Smad2/3 (R&D System, Lille, France), anti-vimentin, anti-Ecadherin, anti-TGFβ1, anti-smad2/3, anti-pSmad2, anti-snail (all from Cell Signaling Technology, Danvers, USA). All antibodies were diluted in Trisbuffered saline and 0.1% (v/v) Tween-20 containing dried milk. Blotted proteins were detected and quantified on a bioluminescence imager and BIO-1D advanced software (Wilber-Lourmat, Marne-la-Vallée, France), after incubating blots with a horseradish peroxidaseconjugated appropriate secondary antibody (Beckman Coulter, Paris). For some experiments, cytoplasm and nuclear subcellular fractions were harvested after differential centrifugation in adapted buffers; the presence or absence of subcellular specific proteins (such as β-actin or histone H1) attested to subcellular separation.

### Co-immunoprecipitation analysis

Cells were lysed in PBS containing 200 mM of TRIS-Hcl pH 7.4, 100 mM Nacl, 1% v/v tritonX100, 1 mM DTT, 15 mM EGTA, 1 mM NaF, 1 mM vanadate and one complete Mini protease Inhibitor Cocktail Tablet (Complete Mini EDTA Free, per 10 mL of lysis buffer, Roche, France). Rabbit anti-TGFRI polyclonal antibodies (Santa-Cruz Biotechnology) and Rabbit IgG polyclonal antibodies were added to the lysates at a final dilution of 1/100 and incubated overnight at 4°C. Protein-G magnetic beads (Invitrogen, Cergy-Pontoise, France) were added to the mix and incubated for additional two hours at 4°C. Proteins were then eluted by magnetic separation and denaturated. Western-Blot was then performed using anti-NRP2 antibody (Santa Cruz Biotechnology, Heidelberg, Germany).

### Histopathologic Analysis and Immunohistochemical Staining of Tissues

Tissue samples, obtained from xenografts were fixed in 4% formalin and paraffin embedded. Then, blocks were cut serially at 4-µm thickness. HES (Hematoxyline Eosine Safran) staining was used to assess morphology. A standard immunohistochemical technique was performed using a Ventana BenchMark XT (Ventana Medical Systems, Inc., Tucson, Arizona) immunostainer with the following primary antibodies: anti E-cadherin (Zymed, San-Francisco, USA), anti-cytokeratin-20 (Zymed, San-Francisco, USA).

### Surface Plasmon Resonance analysis

Design and fabrication of homemade chips compatible with SPR (Surface Plasmon Resonance) have been performed as previously published with the help of the MIMENTO technological platform, Besançon, France [25]. The NRP2 chips fabricated in this study consist in the covalent grafting of Fc-NRP2 entities on chemically activated self assembled monolayer following the procedure of protein chip building recently published [26]. Fc-NRP2 were from RD System (Lille, France). This procedure leads to a coverage of 7.4 +/− 0.1 femtomole/mm^2^ of NRP2. Injections of BSA (Control −), VEGF (Control +) and TGFβ1 are performed at 250 nM in PBS-Tween 0.05%, pH 7.4. Biacore experiments were performed with the Biacore 2000 apparatus at 25°C with a flow rate comprise between 2 and 30 µl/min.

### Real Time-quantitative PCR (RT-qPCR)

Total RNA were extracted using Kit RNeasy mini (Qiagen, Courtaboeuf, France) and reverse transcribed using random hexamers and Moloney murine leukemia virus reverse transcriptase (Life Technologies, Rockville, MD, USA). Duplicate samples were subjected to RT-qPCR. mRNA were quantified using primers listed below: NRP2 (Hs00187290_m1), Snail1 (Hs001955991_m1), TGF-β1 (Hs00171257_m1), Gli1 (Hs00171790_m1), Twist1 (Hs00361186_m1) (Applied Biosystems).

ABL mRNA from each sample was quantified as an endogenous control of internal RNA. Relative mRNA expression was calculated using the Delta-Delta-Ct method and untreated cells were used as calibrator.

### Slide preparation and confocal microscopy

Cells were spread onto Labteck chamber slides (Sigma-Aldrich, Lyon, France) and subsequently treated with the appropriate reagents. Cells were fixed in 4% paraformaldehyde and permeabilized with 0.1% Triton X100. After 20 minutes of blocking in 20% fetal bovine serum and washing, cells were stained with appropriate antibodies Stacks of confocal images were collected with an Olympus FV1000 laser scanning confocal microscope. Cell nuclei were counterstained with DAPI (4′,6-diamidino-2-phenylindole). For fluorescence quantification, ratio of fluorescence intensity was calculated in each condition. Ratio of fluorescence intensity was calculated in dividing the nuclear fluorescence by the cytoplasm-membrane fluorescence. Fluorescence intensity of 50 cells has been analyzed in each condition.

### Smad reporter assay

We have used the TGF-β reporter assay from SABiosciences (Qiagen, Courtaboeuf, France) for the quantification of TGF-β-induced SMAD2/3 signaling. Quantification of the firefly luciferase was realized using a Dual-Luciferase Reporter assay system (Promega Co, Madison, USA) according to the manufacturer's protocol. All transfections were performed in triplicate using Lipofectamine kit. (Invitrogen, Cergy-Pontoise, France). After 24 h of transfection, medium was changed and cells were treated by 50 ng/mL of TGF-β1 for additional 24 hours. Results are presented as the ratio between the *firefly luciferase* activity and the *renilla luciferase* activity (Ren/Luc) for each conditions. Then, values were reported to the values of the negative control.

### Statistical analysis

Results are expressed as the mean plus or minus the standard error of the mean (SEM). Group comparisons were performed using Student *t* test. Differences were considered significant at *p*<0.05.

## Results

### NRP2 expression in human cancer cell lines

**Figure 1:**
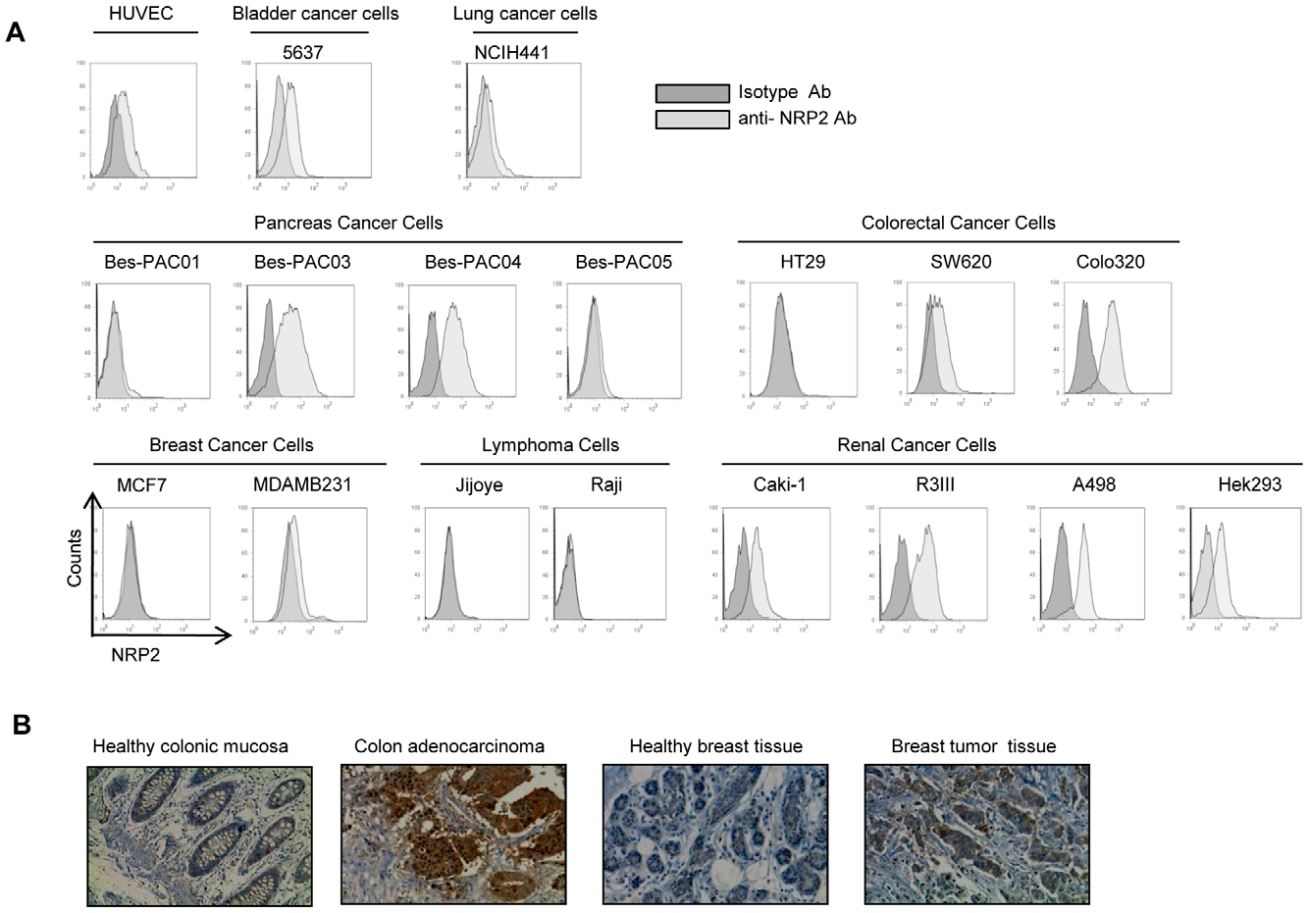
Neuropilin-2 expression on human cancer cells. *A,* Flow cytometry analysis of NRP2 expression in human cancer cell lines. *B,* Immunohistochemical staining of NRP2 in human colon tissues and breast tissues (brown). Formalin-fixed paraffin-embedded tissues were incubated overnight at room temperature with anti-human NRP2 antibody. Representative micrographs were taken at an original magnification x1000; NRP2 is expressed at the membrane of human colon and breast carcinomas while it is not expressed in healthy tissues.

We first sought to examine the expression of NRP2 glycoprotein in various cancer cell lines. Immunofluorescence analysis confirmed that NRP2 is expressed at the membrane of several human cancer cell lines (Figure 1A). Human umbilical vein endothelial cells (HUVEC), isolated from normal human umbilical vein, were used as a positive control for NRP2 expression. We observed NRP2 expression at the membrane of 2 out of 3 colon cancer cell lines (SW620, Colo320 but not HT29). NRP2 was also expressed in all renal cancer cell lines tested (HEK 293, Caki, R3III and A498), in two of four pancreatic cancer cell lines (Bes-PAC03 and Bes-PAC04, derived from patient's ascitic fluid in our institute), in NCIH441 lung cancer cell line and in 5637 bladder cancer cell line (Figure 1A). MDAMB231 breast cancer-cell line expressed NRP2 whereas no NRP2 staining was found on Burkitt lymphoma cells lines (Raji, Jijoye) (Figure 1A).

Immunohistochemistry studies were then undertaken to determine if NRP2 is expressed at the membrane of various paraffin embedded-human cancer specimen. NRP2 was expressed on 3 out of 10 colon carcinoma, 5 out of 15 breast carcinoma and 4 out of 12 pancreatic carcinoma. Of note, NRP2 was not detected on prostate cancers (n = 10) and B cell lymphoma (n = 10) (data not shown). Moreover, immunohistochemical staining showed that NRP2 is expressed at the membrane of human colon carcinoma and breast carcinoma while it is not expressed in non malignant tissues (Figure 1B). Our results are concordant with previous published reports. Indeed, in a recent study, Gray et al observed that NRP2 was not detectable in nonmalignant colonic mucosa but was evident in 10 (83%) of 12 adjacent colon adenocarcinoma and in five (71%) of seven liver metastases by IHC staining. Moreover, in another study, NRP2 expression was found in 5 out of 6 (83%) commonly used pancreatic cell lines [15] and in 7 out of 11 (64%) surgical specimens of pancreatic adenocarcinoma by IHC staining [14]. Finally, in breast cancer, Yasuoka et al reported Nrp2 expression in 60 out of 113 invasive breast carcinoma (53.1%) [18]. From these various studies, it appears that NRP2 seems to be specific of several tumor tissues, while no expression of this glycoprotein is commonly observed in healthy tissues, confirming that NRP2 is an attractive target for innovative anti-tumor therapies (see Table S1 for review of NRP2 expression in cancers).

**Figure 2:**
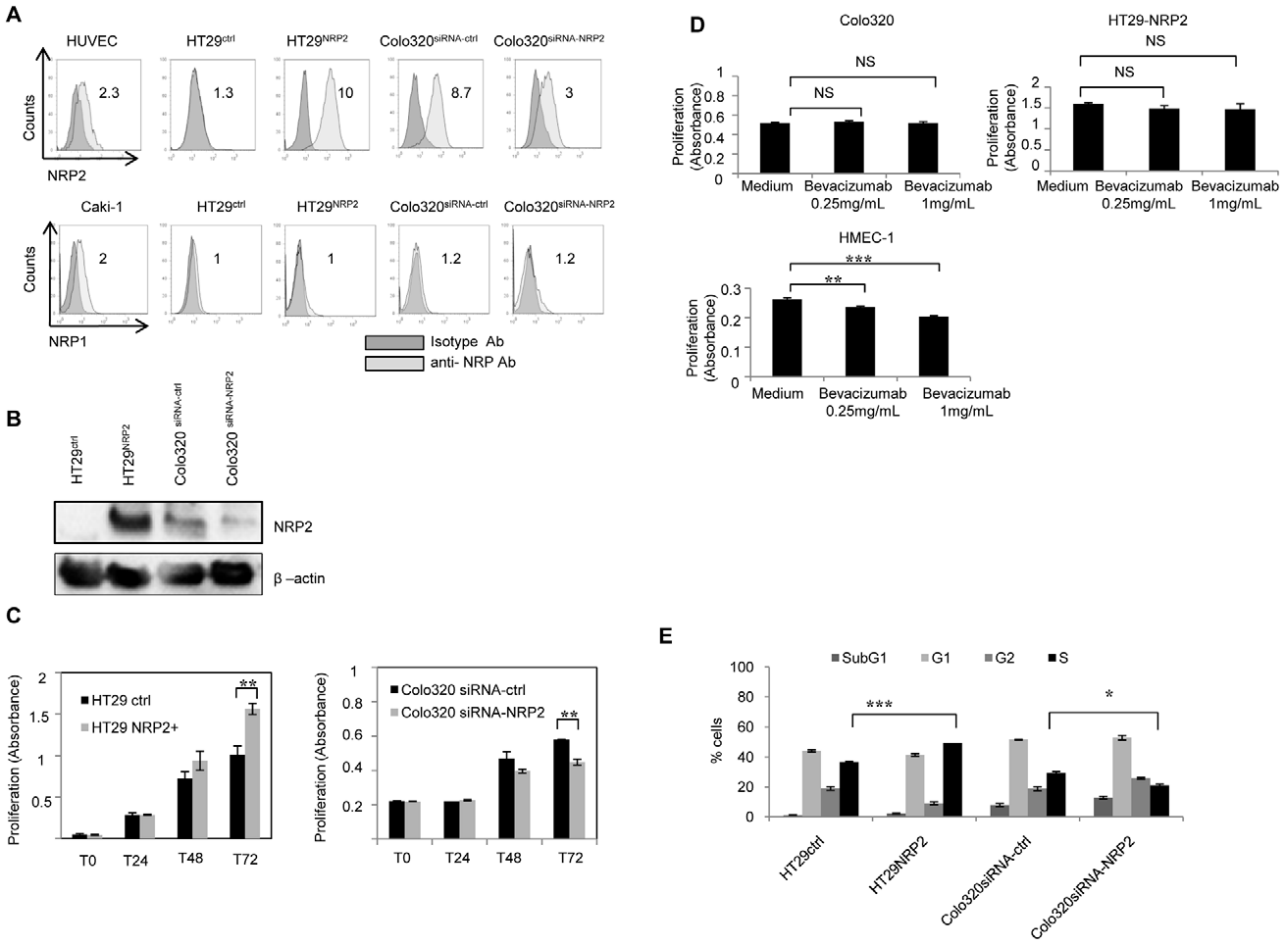
Neuropilin-2 promotes cell proliferation in colon carcinoma cell lines. Transfected cells were analyzed for NRP1 and NRP2 expression by flow cytometry analysis (*A*) or by western blotting (*B*). For flow cytometry analysis, Relative Fluorescence Intensity (RFI) was calculated. Caki1 and HUVEC cells were used as positive control for NRP1 and NRP2 staining respectively. *C,* Proliferation of HT29 and Colo320 cells, according to NRP2 expression was assessed using MTT assays. 4000 cells were let in culture during 24, 48 or 72h before analysis. NRP2 expression is associated with an enhanced proliferation in colon cancer cells. Data represent means of triplicates plus or minus the standard error (SE) of a representative experiment out of three performed. (**, P<0.01). D, Similar MTT experiments were reproduced in the presence of bevacizumab (0.25 and 1 mg/mL, 72 h). HMEC-1 microvascular endothelial cells were used as a positive control for the bioactivity of bevacizumab. Indeed, bevacizumab significantly decreases HMEC-1 proliferation whereas no decrease of cell proliferation is observed with HT29^NRP2^ and Colo320 cancer cells. Experience was made 3 times, and for each time in triplicates. *E,* Flow cytometric analysis of DNA content of transfected colon cancer cells. NRP2 expression is associated with an enhanced number of cells in phase S. Data represent results of a representative experiment out of 3 expressed as the mean of duplicate assays (*, P<0.05; **, P<0.01, ***P<0.001).

To study the precise role of NRP2 in cancer progression, we decided to generate colon cancer cell lines expressing or not NRP2, using NRP2 gene transfer or NRP2 specific siRNA. Hence, NRP2 was transfected into HT29 and a specific siRNA was used to knockdown NRP2 expression in Colo320. Flow cytometry experiments were performed to confirm the modulation of NRP2 expression in HT29 and Colo320 (Figure 2A). No modulation of NRP1 expression was observed in HT29 or Colo320 transfected cells. Caki-1 renal cancer cells were used as positive control for NRP1 staining. NRP2 presence in HT29^ctrl^, HT29^NRP2^, Colo320^siRNA-ctrl^, Colo320^siRNA-NRP2^ was controlled by western blotting (Figure 2B).

### NRP2 promotes tumor proliferation

We took advantage of the previous cell lines to study the role of NRP2 on cancer proliferation in vitro and tumor growth in vivo. Proliferation was monitored using MTT assays. HT29^NRP2^ cells showed a superior proliferation rate at 24, 48 and 72 h compared to HT29^ctrl^ (Figure 2C). Conversely, NRP2 knockdown using specific siRNA, negatively modulated the proliferation of Colo320 tumor cell line (Figure 2C). These experiments showed that NRP2 expression enhances colon cancer cell line proliferation in vitro (the significativity at each time point of these MTT assays is indicated in Table S2). To confirm the influence of NRP2 on cell proliferation, we have evaluated in two additional experiments the doubling-times of HT29^ctrl^, HT29^NRP2^, Colo320^siRNA-ctrl^ and Colo320^siRNA-NRP2^ cancer cells. NRP2 expressing cells HT29^NRP2^ and Colo320^siRNA-ctrl^ had a doubling time of 8 and 11 hours respectively, whereas doubling times of NRP2 lacking cells HT29^ctrl^ and Colo320^siRNA-NRP2^ were 13 and 14 hours. Since NRPs are VEGF co-receptors, we monitored VEGF-A production in HT29 and Colo320 cultures by Elisa test. These cells produced low levels of VEGF-A. NRP2 expression did not influence VEGF production (Figure S1). Moreover, the neutralizing mAb bevacizumab, known to inhibit the proliferation of microvascular endothelial cell line HMEC-1 [27], was used to address the potential role of VEGFA in NRP2-mediated tumor cell growth. These experiments showed that VEGFA neutralization did not influence NRP2-mediated HT29 or Colo320 proliferation. (Figure 2D)

Moreover, NRP2 is a functional receptor for semaphorin 3F, which was described as an inhibitor of angiogenesis, tumor progression and metastasis [28]. Western blotting experiments showed that HT29^ctrl^ and HT29^NRP2^ express the same level of semaphorin 3F, whereas no semaphorin 3F was found in Colo320 cells, suggesting that NRP2-mediated tumor proliferation does not involve semaphorin 3F (Figure S2). Then, the distribution of the nuclear DNA content was studied by flow cytometry in HT29^ctrl^, HT29^NRP2^, Colo320^siRNA-ctrl^ and Colo320^siRNA-NRP2^ cancer cells. NRP2 expression was associated with an enhanced number of cells in phase S (Figure 2E). Serum deprivation in culture medium induced an increase in the subG1 fraction only in NRP2 negative conditions (data not shown). Collectively, these results showed that NRP2 expression in colorectal cell carcinoma promotes cancer cell proliferation and survival.

### NRP2 ablation using siRNA inhibits xenograft formation

**Figure 3:**
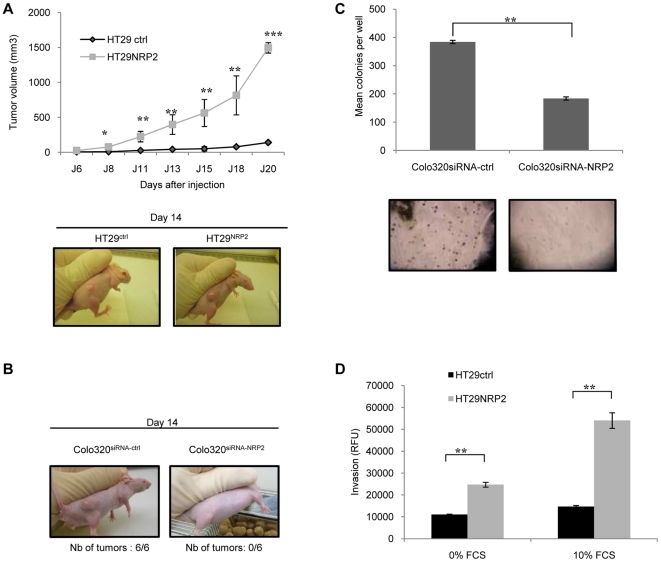
NRP2 enhances colorectal cancer xenograft formation. *A,* 1.10^6^ HT29^ctrl^ and HT29^NRP2^ cells were injected subcutaneously in nude mice (15 mice in each group included in four independent experiments). NRP2 significantly enhanced tumor growth of colon cancer cells in xenograft experiments. Tumor volume was calculated by the formula *V* = 1/2 *a* × *b* ^2^, where *a* is the longest tumor axis, and *b* is the shortest tumor axis. Data reported are the mean average tumor diameter ± SEM. A representative experiment out of 3 is shown. (**, P<0.01). B, 1.10^6^ Colo320^siRNA-NRP2^ or Colo320^siRNA-ctrl^ were injected s.c in nude mice (15 mice in each group included in four independent experiments). Mice receiving Colo320^siRNA-NRP2^ did not develop tumor, even after 2 months. Data represent results of a representative experiment out of 3. *C,* 5000 Colo320 cells treated with control siRNA or NRP2 siRNA were cultured in soft Agar containing medium during 10 days. Colonies were then photographed (magnitude*10) and counted. SiRNA-NRP2 prevents colony formation in soft agar assays, in comparison to siRNA-ctrl in Colo320 cancer cells. (**, P<0.01). D, Invasion assays were performed using HT29^ctrl^ and HT29^NRP2^. HT29^NRP2^ were resuspended in serum-free medium and placed in the top compartment of a standard 8 µM pore Boyden chamber. Following 16 hours of culture (37°C, 5% CO2), invasive cells were incubated with cell detachment buffer, lysed and marked with CyQuant GR dye. Fluorescence was then evaluated with a fluorescence plate reader using a 480-520 nm filter set.

The precise role of NRP2 on cancer progression was first characterized *in vivo*. To examine the effect of NRP2 expression on *in vivo* tumor growth, we inoculated equal numbers (1.10^6^ cells per mouse) of HT29^NRP2^ or HT29^ctrl^ subcutaneously into nude mice. Tumor incidence and volume were assessed biweekly. Tumors appeared in all mice inoculated with HT29^ctrl^ and HT29^NRP2^. NRP2 significantly enhanced tumor growth in vivo (Figure 3A). To confirm these results, we decided to investigate if NRP2 targeting using specific siRNA could inhibit tumor formation. While 1.10^6^ Colo320^siRNA-ctrl^ cells injected subcutaneously into nude mice induced tumor engraftment in all mice, NRP2 inhibition using specific siRNA prevented Colo320 engraftment in all animals suggesting a critical role of NRP2 in the early events contributing to tumor formation (Figure 3B).The influence of NRP2 inhibition using specific siRNA on Colo320 tumorigenicity was confirmed *in vitro*. For this purpose, Colo320 cells were treated with NRP2 siRNA or ctrl siRNA and cultured in a soft agar assay. NRP2 knockdown in Colo320 decreased the number of colonies observed in soft agar experiments (Figure 3C). Since HT29 did not form colonies in soft agar cultures, we decided to investigate the influence of NRP2 on HT29 invasion and migration. For this purpose, a Boyden chamber assay was performed to quantify HT29 invasiveness according to NRP2 expression. The capacity of HT29 cells to migrate through the matrigel-coated filter was significantly enhanced in the presence of NRP2 (Figure 3D). Similar results were observed with Colo320 (data not shown). Of note, these results were also reproduced in the absence of serum suggesting an autonomous effect of NRP2 on the invasive properties of HT29 cells.

### NRP2 induces epithelial-mesenchymal transition

The cell autonomous effect of NRP2 on HT29 invasiveness led us to explore the direct impact of NRP2 on EMT. The phenomenon of EMT is defined by the transition of epithelial cells to fibroblastoid- or mesenchymal-like cells. EMT is an important mechanism associated with cancer invasiveness and metastasis formation. In a recent paper, NRP1 was found to drive EMT process by promoting Snail1 nuclear localization in prostate cancer cells [29]. In culture, NRP2 transfection conferred to HT29 a fibroblastic-like shape reminiscent of mesenchymal cells, whereas HT29 and HT29^ctrl^ cell lines displayed a cuboidal appearance, formed clusters which progressively increased in number, and closely apposed cell-to-cell junctions typical of epithelial cells (Figure S3A). This observation prompted us to investigate the ability of NRP2 to orchestrate the EMT.

**Figure 4:**
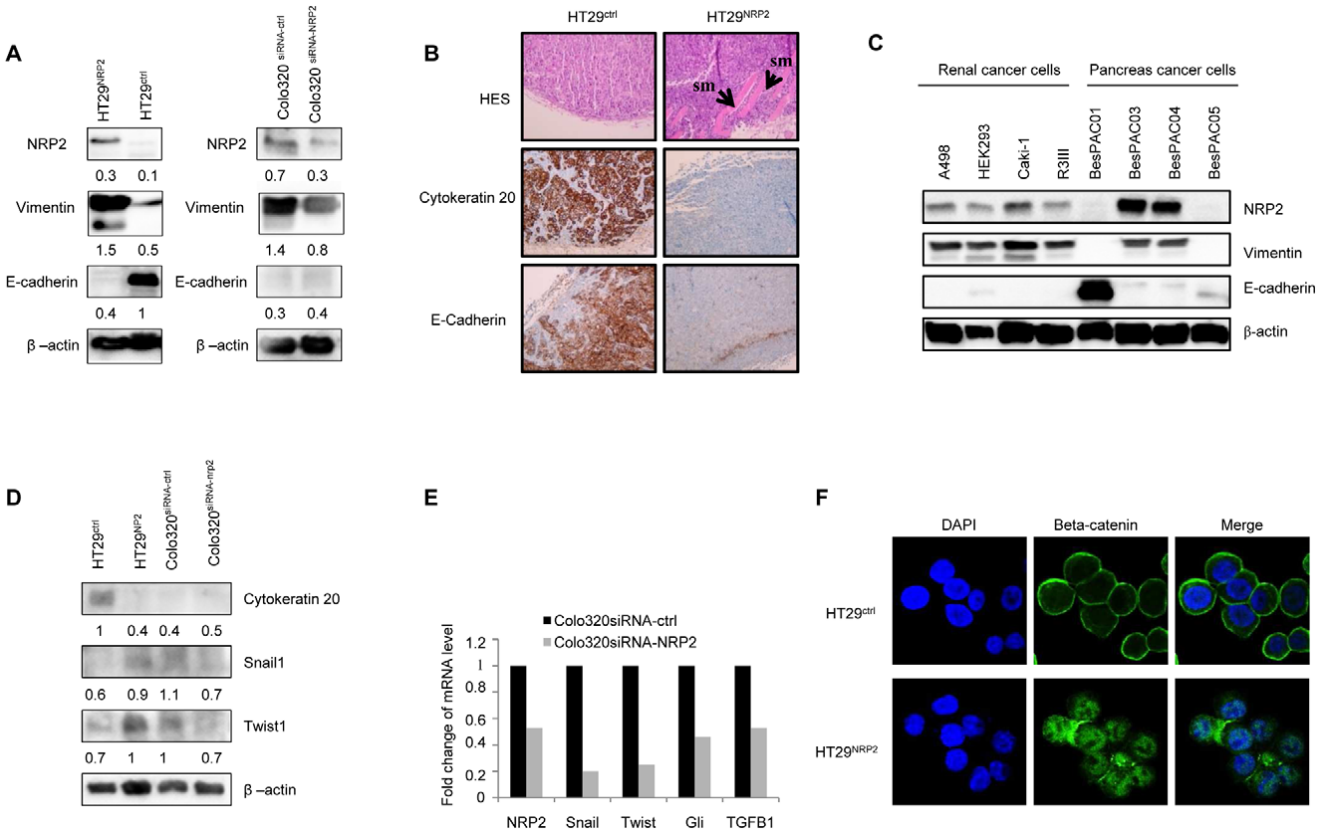
NRP2 drives EMT in colon cancer cells. *A,* HT29^ctrl^, HT29^NRP2^, Colo320^siRNA-NRP2^ and Colo320^siRNA-ctrl^ were analyzed for expression of epithelial and mesenchymal markers (respectively E-Cadherin and vimentin) by western-blotting (whole cell lysate). β-actin was used as a control of protein loading. The presence of NRP2 decreases E-cadherin expression and induces vimentin in HT29 colorectal cancer cells. Specific disruption of NRP2 using siRNA down-regulated vimentin in Colo320 cancer cells. Blotted proteins have been quantified with the BIO-1D advanced software and reported to the β-actin level. *B,* Frozen sections of the isolated tumors from HT29^ctrl^ and HT29^NRP2^ xenografts were subjected to immunohistochemical staining with anti-cytokeratin-20, anti-Ecadherin antibodies and HES staining (magnification x100). HES (Hematoxiline Eosine Safran) staining was used to assess morphology. HT29^NRP2^ invade smooth muscle (sm = smooth muscle) as indicated by arrows unlike HT29^ctrl^ which showed a local invasion. HT29^NRP2^ xenografts lacked epithelial markers such as cytokeratin-20 and E-cadherin. *C*, Pancreatic and renal cancer cell lines were analyzed for NRP2, E-cadherin and vimentin expression by western-blotting. β-actin was used as a control of protein loading. The presence of NRP2 is positively correlated with mesenchymal marker vimentin and inversely correlated with epithelial marker E-cadherin. *D*, Transcriptional factors Snail1 and Twist1 were analyzed in HT29^ctrl^, HT29^NRP2^, Colo320^siRNA-NRP2^ and Colo320^siRNA-ctrl^ whole cell lysates by western-blotting. NRP2 expressing cells (Colo320^siRNA-ctrl^ and HT29^NRP2^) have increased levels of Snail1 and Twist1 compared to NRP2 lacking cells (HT29^ctrl^ and Colo320^siRNA-NRP2^). β-actin was used as a control of protein loading. Blotted proteins have been quantified with the BIO-1D advanced software and reported to the β-actin level. *E,* Evaluation of NRP2, Snail, Twist1, Gli1 and TGFβ1 expression by QRT-PCR analysis in Colo320^siRNA-ctrl^ and Colo320^siRNA-NRP2^. Decreased NRP2 expression is indeed associated with a down-regulation of transcriptional regulators Snail, Gli1 and twist1 and with a decreased TGF-β1 production. *F,* Confocal microscopy analysis of localization of β-catenin. Cells (HT29^ctrl^ and HT29^NRP2^) were stained with DAPI (blue) for nuclear staining and also with anti-β-catenin antibody (green). Whereas β-catenin (green) is localized at the membrane in HT29^ctrl^ cells, HT29^NRP2^ cells show a nuclear localization of this transcriptional activator.

EMT is characterized by the loss of epithelial markers and acquisition of mesenchymal components. E-cadherin, occludin and cytokeratin are downregulated during EMT, while N-cadherin, vimentin, fibronectin are upregulated [30]. E-Cadherin is a universal epithelial marker that plays a key role in epithelial integrity maintenance. Loss of its expression is a hallmark of EMT. Vimentin is a canonical marker for detecting fully transitioned epithelial cells and their acquisition of fibroblastoid-like phenotype. Accordingly, we assessed the pattern of E-Cadherin and vimentin expression in colorectal cancer cell lines by western-blotting. The presence of NRP2 in HT29 completely abrogated the expression of E-Cadherin and induced the production of vimentin. (Figure 4A). This observation was confirmed in Colo320 where specific disruption of NRP2 using siRNA down-regulated vimentin (Figure 4A). Then we analyzed the expression of EMT-associated markers in HT29^ctrl^ and HT29^NRP2^ xenografts. Immunohistochemical analysis revealed that the presence of NRP2 on HT29 was associated with an invasive phenotype. While HT29^ctrl^ xenografts reconstituted the morphology of moderate differentiated colon adenocarcinoma, HT29^NRP2^ induced less differentiated xenografts displaying an invasive phenotype (Figure 4B). HT29^NRP2^ xenografts lacked epithelial markers such as cytokeratin-20 and E-cadherin compared to HT29^ctrl^ xenografts (Figure 4B). In order to confirm that NRP2 expression is associated with a mesenchymal phenotype, we have determined NRP2, E-cadherin and vimentin levels in four renal and pancreatic cell lines by western-blotting. We observed that NRP2 expression is correlated with vimentin expression and with a lack of E-cadherin expression in these cancer cell lines. (Figure 4C)

Of note, NRP2 transfection in HT29 also led to the decrease of EpCAM (epithelial cell adhesion molecule) in HT29 cells (Figure S3B). We could also observe an inverse correlation between NRP2 and Epcam in several pancreatic cancer cell lines (Figure S3C), confirming that NRP2 is quite associated with a mesenchymal phenotype of cancer cells. Several zinc-finger transcriptional regulators of EMT can act as E-Cadherin repressors such as Snail, which is the master repressor of E-cadherin [31], but also Twist [32]. Gli family members are zinc-finger transcription factors, which are involved in embryogenesis and carcinogenesis. Hedgehog-Gli signaling axis has been recently proposed as a regulator of EMT process through interactions with TGF-β1 pathway [33]. Then, the next set of experiments was performed to analyze EMT-related transcription factors in HT29 and Colo320 cell lines, according to NRP2 expression. Western-Blot experiments showed an increase of Twist and Snail in HT29^NRP2^ in comparison to HT29^ctrl^. (Figure 4D) Conversely, NRP2 targeting using specific siRNA in Colo320 led to a decreased expression of these transcription factors, as shown in western blotting experiments (Figure 4D). Of note, the influence of NRP2 on EMT-related transcription factors was confirmed at the RNA level. Indeed, expression of these transcriptional regulators of EMT was analyzed by real-time quantitative PCR in Colo320^siRNA-ctrl^ and Colo320^siRNA-NRP2^. Decreased NRP2 expression was associated with a down-regulation of Snail1, Twist1 and Gli1 transcription (Figure 4E). Moreover, since nuclear accumulation of the transcriptional activator β-catenin is a hallmark of EMT in cancer cells [34], we decided to evaluate localization of this protein in HT29^ctrl^ and HT29^NRP2^ by confocal microscopy. Whereas β-catenin is localized at the membrane in HT29^ctrl^ cells, HT29^NRP2^ cells show a nuclear localization of this transcriptional activator. (Figure 4F) Furthermore, TGF-β1 is a Janus-like cytokine and has been demonstrated to play a pivotal role in EMT [35], [36]. The expression of TGF-β1 was then monitored in our various colorectal cancer cell lines. We observed that NRP2 knock-down in Colo320 decreased TGF-β1 production (Figure S4) while NRP2 expression in HT29 was associated to a constitutive production of TGF-β1. (Figure S4) Altogether, these results identified a direct role for NRP2 on EMT promotion in colorectal cancer cells.

### Regulation of TGF-β1 pathway by NRP2

Next, we investigated the influence of NRP2 on TGF-β1 signaling. In response to ligand binding, type I and II TGF-β receptors (TGFRI, TGFRII) form tight complexes leading to phosphorylation of Smad2 and Smad3. Phosphorylated Smads interact with cytoplasmic Smad4 and translocate into the nucleus where Smad complex control transcription of target genes. A recent study suggested that NRP1 can be a co-receptor for both active TGFβ1 and TGFβ1-LAP [37]. Moreover, NRP1 associates with TGFRI and TGFRII to enhance TGFβ1 signaling in cancer cells, augmenting canonical Smad2/3 signaling [38]. A peptide of the b2 domain of NRP1 (RKFK, similar to a thrombospondin-1 peptide, (also present in NRP2) activates the latent form of TGF-β1 [37]. Moreover, in fibroblastic cells, NRP1 upregulates TGFβ1 pathway by enhancing smad2/3 phosphorylation and promotes a myofibroblast phenotype [39]. Then, considering the potent promoter effect of NRP1 on TGFβ-1 pathway and on EMT, we hypothesized that NRP2 might have similar properties.

**Figure 5:**
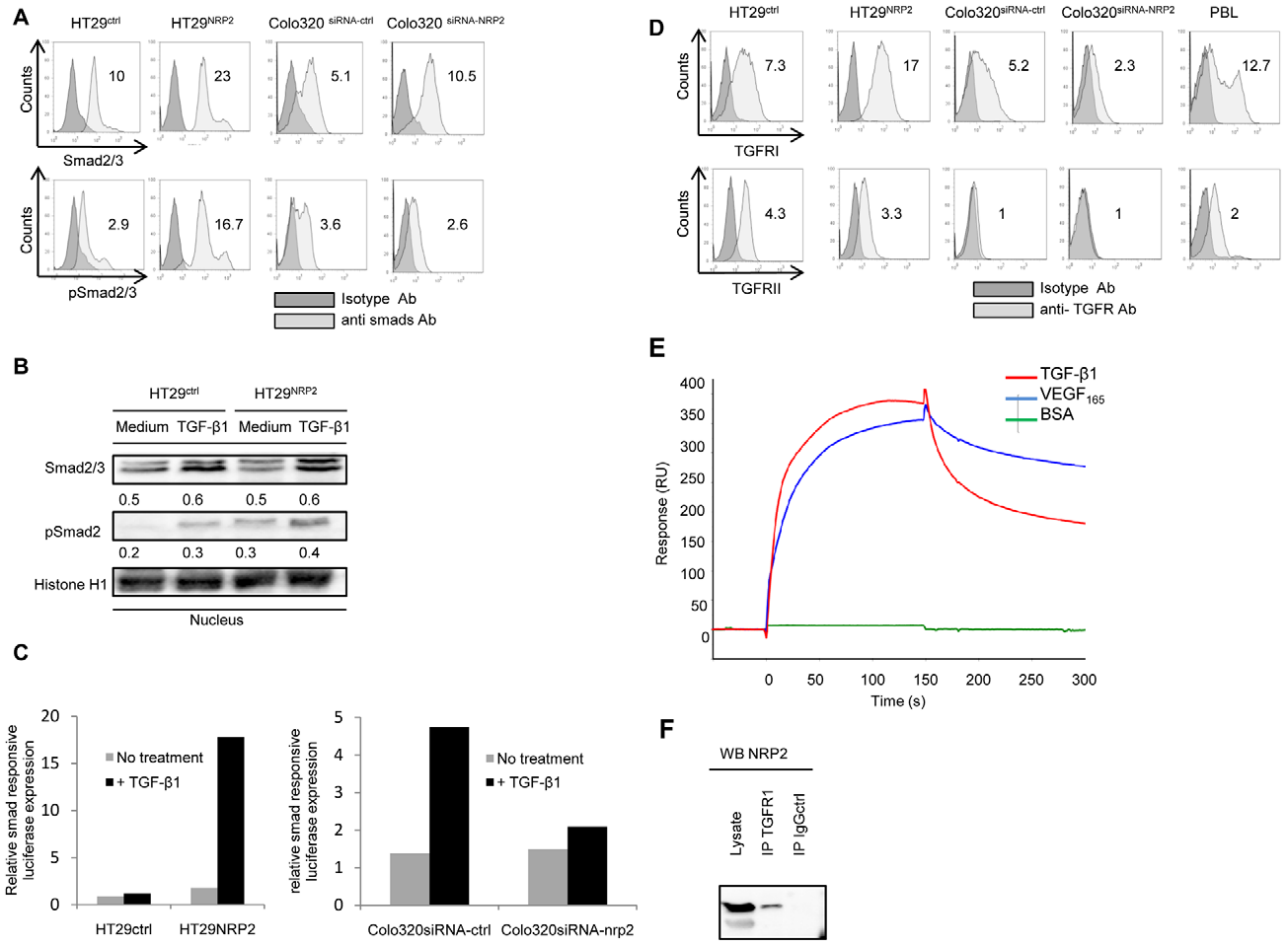
NRP2 acts as a TGF-β1 co-receptor. *A,* smad2/3 and phospho-smad2/3 expression was assessed in HT29^ctrl^, HT29^NRP2^, Colo320^siRNA-ctrl^, Colo320^siRNA-NRP2^ by intra-cellular flow-cytometry. Relative Fluorescence Intensity (RFI) was calculated. *B*, Western-blotting experiments were performed on nuclear extracts of HT29^ctrl^ and HT29^NRP2^ cultured in serum free conditions or with 10 ng/mL of TGF-β1 for 16 hours. Histone-H1 was used as a control of protein loading. Blotted proteins have been quantified with the BIO-1D advanced software and reported to the Histone-H1 level. While exogenous TGF-β1 is mandatory for smad2 phosphorylation detection in HT29, Smad2 is constitutively phosphorylated in HT29^NRP2^ cells without any previous exposition to exogenous TGF-β1. *C,* TGF-β1 Cignal reporter assay kit was used for the quantification of TGF-β1-induced smad2/3 signaling in HT29^ctrl^, HT29^NRP2^, Colo320^siRNA-ctrl^ and Colo320^siRNA-NRP2^ cells. An up-regulation of approximatively 17 times of the smad response is observed in HT29^NRP2^ cells compared to HT29^ctrl^ after TGF-β1 stimulation. At the opposite, smad dependent response is significantly decreased in Colo320 cells treated by siRNA targeting NRP2 compared to Colo320 cells treated by siRNA-control after TGF-β1 stimulation. Results are presented as the ratios between the *firefly luciferase* activity and the *renilla luciferase* activity (Ren/Luc) for each conditions. This experiment was realized 3 times, each time in triplicates. *D,* Expression of TGFRI and TGFRII in HT29^ctrl^, HT29^NRP2^, Colo320^siRNA-ctrl^, Colo320^siRNA-NRP2^ colon cancer cells. Relative Fluorescence Intensity (RFI) was calculated. Colo320 cells don't express TGFRII. *E,* Surface plasmon resonance studies were performed to explore TGF-β1 interactions with NRP_2_. Fc-NRP2 proteins were covalently grafted on a chemically activated self assembled protein chip. Injections of TGF-β1, BSA (Control -), VEGF (Control +) were performed at 250 nM in PBS-Tween 0.05%, before biacore analysis. These experiments were reproduced two times and showed a specific binding of TGFβ1 to Fc-NRP_2_ protein. *F,* NRP2 interacts with TGFRI in co-immunoprecipitaion experiments using HT29^NRP2^ cells.

Hence, we first assessed Smad2/3 activation status according NRP2 expression in HT29 and Colo320. Using flow cytometry and western blot experiments, we observed that NRP2 expression is associated to a constitutive smad2/3 phosphorylation (Figure 5A and B). While exogenous TGF-β1 was mandatory for smad2 phosphorylation detection in HT29 using western blotting experiments, we could observe that Smad2 was constitutively phosphorylated in HT29^NRP2^ cells without any previous exposition to exogenous TGF-β1 (Figure 5B). Moreover, NRP2 expression was associated with an increase of TGF-β1 on HT29 cytoplasmic membrane, while treatment of Colo320 with NRP2-siRNA decreased membrane-bound TGF-β1 (Figure S5). To confirm our hypothesis that NRP2 induces a constitutive activation of the TGFβ1 pathway, we used a TGF-β1 signaling reporter assay for the quantification of TGF-β1-induced smad2/3 signaling in HT29^ctrl^, HT29^NRP2^, Colo320^siRNA-ctrl^ and Colo320^siRNA-NRP2^ colorectal cancer cells. Compared to HT29^ctrl^, HT29^NRP2^ exhibited an enhanced smad2/3 activity, as reported in the Figure 5C. At the opposite, smad responsive luciferase expression is significantly decreased in Colo320 cells treated by siRNA targeting NRP2 compared to Colo320 cells treated by siRNA-control after TGF-β1 stimulation. (Figure 5C) NRP2 exerts a co-stimulatory effect of the TGF-β1 pathway in colorectal cancer cells.

Then, we characterized TGFRI and TGFRII expression on HT29 and Colo320. Interestingly, while the kinase containing receptor, TGFRI, was expressed both on HT29 and Colo320, TGFRII, known to bind TGF-β1, was not present on Colo320 (Figure 5D). Combined with the inhibition of membrane-bound TGF-β1 by NRP2 targeting siRNA (Figure S5), these results supported the hypothesis of a direct binding activity of TGF-β1 by NRP2 leading to smad2/3 phosphorylation in colorectal cancer cells.

To examine if NRP2 could directly bind active TGF-β1, we studied the binding of TGF-β1 to NRP2-Fc by Surface Plasmon Resonance (SPR). We showed that TGF-β1 can directly bind to NRP2-Fc chips as well as VEGF_165_ (Figure 5E). BSA was used as a negative control. Specific Interactions were highlighted by checking the interactions on Fc-control chips (bevacizumab coated chips) (data not shown). These results were confirmed in co-immunoprecipitation experiments, where TGFRI could be detected in NRP2-targeted immunoprecipitation. (Figure 5F) Therefore, these results provide evidences of a direct binding activity of NRP2 for TGF-β1, leading to cooperation with TGFRI and subsequent smad2/3 phosphorylation.

### NRP2-induced EMT is TGF-β1 dependent

**Figure 6:**
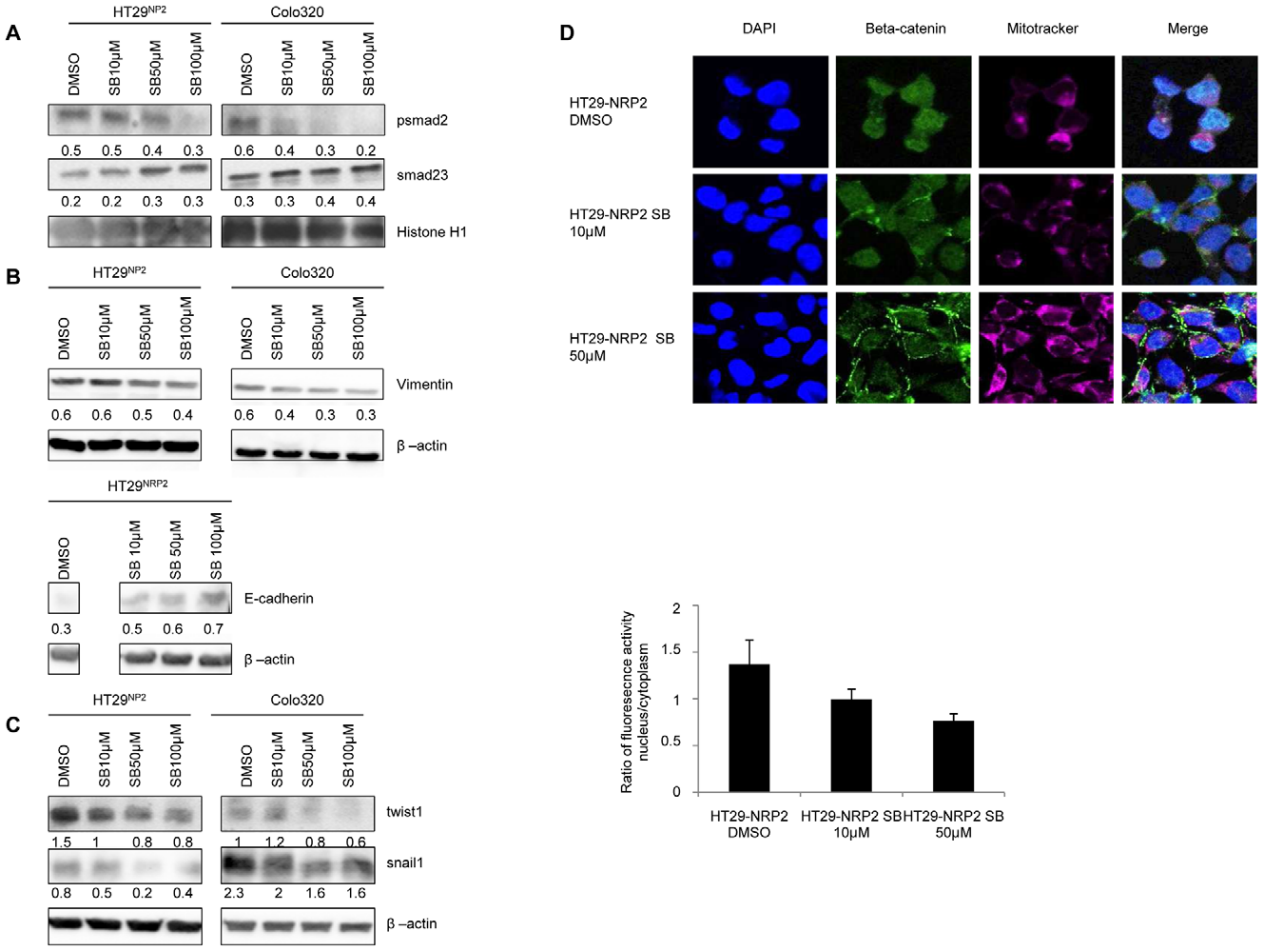
TGF-β1 is required for NRP2-mediated EMT. *A,* Western-blotting experiments were performed on nuclear extracts of HT29^NRP2^ and Colo320 for the detection of nuclear smad proteins after treatment with increased doses of TGFRI kinase-inhibitor SB-431542 (10 µM, 50 µM). DMSO is the control medium. Histone-H1 was used as a control of protein loading. Whereas only 10 µM of SB-431542 decreases the nuclear level of phosphorylated Smad2 in Colo320 cancer cells, doses of 50 µM should be used to decrease the nuclear level of phosphorylated Smad2 in HT29^NRP2^ cancer cells. Smad proteins have been quantified with the BIO-1D advanced software and reported to the Histone H1 level. *B*, Overnight treatment with increased doses of TGFRI kinase-inhibitor SB431542 (10 µM, 50 µM, 100 µM) inhibited dose-dependently vimentin expression in Colo320 and in HT29^NRP2^ cells. TGF-β1 signaling inhibition restored in part E-cadherin in HT29^NRP2^ cells. Proteins have been quantified with the BIO-1D advanced software and reported to the β-actin level. Data represent results of a representative experiment out of 3. *C,* Western-blotting experiment realized with Colo320 and HT29^NRP2^ whole cell lysates overnight treated with increased doses of TGFRI kinase-inhibitor SB431542 (10 µM, 50 µM, 100 µM). SB431542 treatment inhibited dose-dependently snail1 and twist1 expression in Colo320 and in HT29^NRP2^ cells. Proteins have been quantified with the BIO-1D advanced software and reported to the β-actin level. *D*, Confocal microscopy analysis of localization of β-catenin in HT29^NRP2^ cells after overnight treatment with increased doses of TGFRI kinase-inhibitor SB431542 (10 µM, 50 µM). Cells (HT29^NRP2^) were stained with DAPI (blue) for nuclear staining, with mitotracker (purple) for cytotoxicity dectection and also with an anti-β-catenin antibody (green). Mitotracker staining (purple) was realized in order to assess potential cytotoxicity of the SB-431542 treatment. Loss of staining of this marker correlates with increased cytotoxicity. 16 hours treatment with 50 µM of SB-431542 induces relocalization of beta-catenin at the membrane of the cells, indicating that TGFRI activity neutralization by SB-431542 can reverse EMT in HT29^NRP2^ colorectal cancer cells. No cytotoxicity was observed at any dose of SB-431542 used. For fluorescence quantification, ratio of fluorescence intensity was calculated in each condition. A decreased ratio of nuclear fluorescence/cytoplasm-membrane fluorescence intensity after treatment with 50 µM of SB-431542 in HT29^NRP2^ cells was observed indicating that beta-catenin relocalizes at the membrane of the cells.

Since NRPs are non-tyrosine kinase receptor, NRP2 and TGFRI are thought to cooperate in order to mediate smad phosphorylation and subsequent induction of EMT. Then, the next set of experiments was performed to examine if TGF-β1 pathway neutralization in NRP2 positive cells can reverse NRP2-mediated EMT. For TGF-β1 pathway neutralization, we used two pharmacological inhibitors of TGFRI, SD-208 and SB-431542. SD-208 and SB-431542 are specific inhibitors of TGF-β superfamily type I receptors [40], [41], [42]. Treatment of NRP2 expressing cells with variable doses of SB-431542 has been performed to determine the concentrations of this inhibitor required to decrease nuclear smad phosphorylation in HT29^NRP2^. After sub-cellular separation, levels of nuclear phosphorylated smad2 and nuclear total smad2/3 proteins were evaluated by western blotting. Whereas only 10 µM of SB-431542 decreases the nuclear level of phosphorylated Smad2 in Colo320 cancer cells, 50 µM of TGFRI inhibitor decreased the nuclear level of phosphorylated Smad2 in HT29^NRP2^ cancer cells. (Figure 6A)

Then, we assessed the influence of TGFRI pharmacological inhibition on vimentin and E-cadherin levels in NRP2 expressing colorectal cancer cell lines. SB-431542 treatment decreased dose-dependently vimentin expression in Colo320 and HT29^NRP2^ cancer cells and even restored E-Cadherin levels in HT29^NRP2^ underlying that TGFRI plays an essential role in the maintenance of the EMT phenotype conferred by NRP2 (Figure 6B). However, TGFRI inhibition had no influence on E-Cadherin expression in Colo320 which constitutively not express this molecule. Moreover, we could observe a decrease of Snail and Twist transcription factor levels following exposition of HT29^NRP2^ and Colo320 to the TGFRI pharmacological inhibitor SB-431542 (Figure 6C). Then, since nuclear localization of beta-catenin is frequently observed in tumor cells undergoing EMT [43], we analyzed localization of this transcriptional activator in HT29^NRP2^ cells, after treatment by increased doses of SB-431542, by confocal microscopy analysis. A treatment with 50 µM of SB-431542 during 16 hours induced the relocalization of beta-catenin at the membrane of tumor cells, indicating that TGFRI activity neutralization can reverse EMT in NRP2 expressing colorectal cancer cells. (Figure 6D) Mitotracker staining was realized in order to assess potential cytotoxicity of the SB-431542 treatment. No cytotoxicity was observed at any dose of SB-431542 used. For fluorescence quantification, ratio of fluorescence intensity was calculated in each condition. We observed a decrease of the ratio of nuclear fluorescence/cytoplasm-membrane fluorescence intensity after treatments with 50 µM of SB-431542 in HT29^NRP2^ cells, confirming the relocalization of beta-catenin at the membrane of the cells. (Figure 6D).

Moreover, we analyzed the effect of TGFRI kinase inhibition on colony formation in soft agar experiments. For this purpose, Colo320 expressing NRP2 (Colo320^siRNA-ctrl^) or treated by siRNA targeting NRP2 (Colo320^siRNA-NRP2^) were cultured in soft agar with or without SD208, another pharmacological inhibitor of TGFRI and in the presence or not of TGF-β1. The ability of NRP2 to promote the formation of Colo320 colonies in soft agar cultures was not observed in the presence of TGFRI pharmacological inhibition, in presence or not of TGF-β1 (Figure S6). Similar results were obtained with SB-431542 (data not shown). Moreover, exposition to 10 ng/mL of TGF-β1 increased the number of colonies of Colo320^siRNA-ctrl^, whereas this treatment had no impact on Colo320^siRNA-NRP2^ cells. (Figure S6) These experiments suggest that the EMT program mediated by NRP2 depends on TGF-β1.

Altogether, these results suggest that NRP2 expression leads to TGF-β1 constitutive signaling activation and sustains a possible role of NRP2 and TGF-β1 in EMT. The oncogenic properties of NRP2 and its direct involvement in EMT open new perspectives to design specific targeted therapeutics.

## Discussion

There is increasing evidence that epithelial-mesenchymal transition (EMT) is involved in cancer progression. For most carcinomas, cancer progression is correlated with a loss of epithelial differentiation and a shift towards a mesenchymal phenotype. This process, referred to as EMT, enhances motility and invasiveness of many cell types and is often considered as a prerequisite for tumor infiltration and metastasis. The targeting of specific molecules involved in EMT is of particular interest to prevent cancer progression. However, such specific therapies interfering with the EMT process are still missing. Neuropilins were initially described as kinase-deficient neuronal patterning receptors recognizing class-3 semaphorin ligands. Indeed, neuropilins have emerged as regulators of neuronal growth cone collapse and cell motility mediated by semaphorins during embryogenesiss [1] leading us to explore the hypothesis of their involvement in EMT.

Their ability to function as VEGFR co-receptors resulted in the identification of their implication in vascular and lymphatic sprouting controlled by VEGF. The role of NRPs in vascular development regulation supported their potential involvement in cancer neo-angiogenesis. Blocking NRP1 in mice exposed to cancer cells, resulted in a vascular-specific anti-tumor activity [44]. Based on the ability of NRP2 to bind VEGF-A, C and D, studies were conducted to investigate the role of NRP2 in cancer neovascularization. Neutralization of NRP2 in different tumor models led to tumor lymphangiogenesis inhibition [5].

In addition to their expression on neovessels, NRPs have been detected on several human tumors. The wide expression of NRPs among different human tumors suggested a potential role of this molecular network in cancer progression. Although the role of NRP1 in cancer was largely demonstrated [45], the precise contribution of NRP2 in oncogenesis was only recently considered. A role for NRP2 in cancer cells has been initially suggested in colon cancer. The first investigations showed that NRP2 is expressed on human colon cancer cells while undetectable in uninvolved adjacent mucosa [14]. NRP2 expression was shown to promote the malignant phenotype of colon cancers [14], pancreatic cancers [15], and breast cancers [18]. Particularly, NRP2 knock-down hampered anchorage-independent growth in several tumor models [14], [15].

Our results first confirmed the role of NRP2 in cancer proliferation *in vitro* and xenograft formation *in vivo* (Figures 2 and 3). We also extended the understanding of NRP2 oncogenic functions. We have shown that NRP2 is a critical component of epithelial to mesenchymal transition. Analysis of NRP2 transfected cell lines and NRP2 expressing xenografts established that NRP2-expressing tumor cells displayed an immunohistochemical phenotype of EMT characterized by the loss of E-Cadherin and an increase of vimentin expression (Figure 4).

EMT is a pivotal component of cancer invasiveness associated with loss of epithelial differentiation and gain of mesenchyme-like capabilities. Loss of homophilic cell adhesions and polarity is widely associated with loss of functional E-cadherin and acquisition of proteins regulating cytoskeletal functions such as vimentin. A direct role of vimentin in cancer invasiveness, when co-expressed with keratins, was suggested in breast carcinoma cell lines [46]. The expression of vimentin in invasive colorectal carcinoma was not thoroughly investigated. Brabletz T *et al* observed an expression of vimentin in colorectal cancer cell lines and dedifferentiated human colorectal carcinoma [47]. The association between vimentin and EMT in colorectal cancer cell was confirmed in a recent study [48]. Of note, these authors demonstrated that TGFβ-1-mediated EMT directly drives vimentin production in colon cancer cells [48].

However, in our study, treatment with recombinant TGF-β1 failed to induce vimentin expression or to decrease E-cadherin level in HT29 cells (data not shown). In contrast, NRP2 transgenic expression induced vimentin and repressed E-Cadherin in HT29. Moreover, TGFRI pharmacological inhibition restored E-Cadherin expression, suggesting cooperation between TGFRI and NRP2 signaling for EMT promotion. Co-immunoprecipitation experiment confirmed that NRP2 interacts with TGFRI in colon cancer cells. Furthermore, from these results it is postulated that NRP2 expression might be an early component of EMT promoting cadherin switch and acquisition of vimentin expression.

From our results it also appears that NRP2 induction is correlated to the transcription of the E-Cadherin repressors snail and twist1, suggesting a direct role of NRP2 in EMT program acquisition (Figure 4).

Moreover, a peptide (RKFK) of the b2 domain of NRP1, was recently shown to be implicated in TGF-β1 binding [37]. Comparison of the amino acid sequences of NRP1 and NRP2 revealed that RKFK peptide is a conserved sequence of the extracellular part of these receptors.

These observations prompted us to investigate the role of NRP2 on TGF-β1 signaling. Our work highlights a cross-talk between NRP2 and TGF-β1 signaling to promote cancer progression. We could observe that NRP2 gene transfer induces a constitutive expression of activated Smad2/3 and a nuclear localization of phospho-Smad2/3 complexes in HT29 (Figure 5A and 5B). Moreover, NRP2 expression on colon cancer cell lines promoted their capacities to respond to TGF-β1 (Figure 5).

Treatment of Colo320 cell lines with SD208 or SB431542, two TGFR1 kinase inhibitors, abrogated the effect of NRP2 on proliferation in colony formation in soft agar assays (Fig S6). Moreover, TGFRI-kinase inhibitors treatment decreases dose-dependently NRP2-induced vimentin and restores in part E-Cadherin in HT29^NRP2^ cells, suggesting the requirement of TGFRI in NRP2-induced EMT (Figure 6).

The precise role of the TGF-β1-activated Smads in EMT is complex. While activated Smad2/3 complexes promote EMT, the inhibitory Smad7 prevents the transition of cancer cells towards a mesenchymal phenotype [49]. Interestingly, we observed a reduced level of Smad7 in colon cancer cells following NRP2 gene transfer (data not shown). This molecular feature might account for the enhanced colony and xenograft-formation capacities associated with NRP2 expression.

A critical function in cancer cell survival was previously attributed to NRP2 in colon cancer cells [14]. In this model, NRP2 sustains VEGFR1 activity leading to constitutive phosphorylation of Akt and VEGF-mediated survival. Our results suggest the presence of an alternative signaling pathway governed by NRP2 and triggering the Smad-dependent TGF-β1 signaling to initiate the EMT process.

Since EMT is a critical step towards invasion and cancer progression, these results suggest that NRP2 fulfills all the criteria of a therapeutic target to disrupt multiple oncogenic functions in solid tumors.

## Supporting Information

Figure S1 **VEGF-A production was monitored by ELISA assay in HT29^ctrl^, HT29^NRP2^, Colo320^siRNA-NRP2^ and Colo320^siRNA-ctrl^ cells.** Low level of VEGF-A was detected and no significative difference in secretion of VEGF-A was noticed in all cell lines analyzed. NRP2 expression did not influence VEGF production.
(TIF)

Figure S2 **Western blotting experiments showed that HT29^ctrl^ and HT29^NRP2^ express the same level of semaphorin 3F, whereas no semaphorin 3F was found in Colo320 cells.** β-actin was used as a control of protein loading.
(TIF)

Figure S3 ***A,*** **HT29^ctrl^ and HT29^NRP2^ were cultured and observed using a light microscope.** Cells were photographed at magnification *40. HT29^NRP2^ displayed a fibroblastic-like shape whereas HT29^ctrl^ exhibited a cuboidal phenotype. *B*, FACS analysis reveals that NRP2 expression induces decrease of Epcam protein in HT29^NRP2^ cells. *C,* FACS analysis of NRP2 and Epcam proteins in pancreatic cancer cell lines. NRP2 and Epcam are inversely correlated. (red: isotype, green: anti-NRP2 or anti-Epcam antibodies).
(TIF)

Figure S4 **Western-blotting experiments shows that NRP2 positive cells (Colo320^siRNA-ctrl^ and HT29^NRP2^) secreted higher level of TGFβ1 precursors than NRP2 negative cells (HT29^ctrl^ and Colo320^siRNA-NRP2^).** β-actin was used as a control of protein loading.
(TIF)

Figure S5 **The level of membrane associated-TGF-β1 was assessed by FACS analysis in HT29^ctrl^, HT29^NRP2^, Colo320^siRNA-NRP2^ and Colo320^siRNA-ctrl^.** Relative Fluorescence Intensity (RFI) was calculated. NRP2 expression is associated with an increase of TGF-β1 on HT29 cytoplasmic membrane, while treatment of Colo320 with NRP2-siRNA decreased membrane-bound TGF-β1.
(TIF)

Figure S6 **Colo320^siRNA-ctrl^ or Colo320^siRNA-NRP2^ were cultured in soft-agar containing medium for 10 days with or without TGFRI pharmacological inhibitor (SD208, 10µMol).** DMSO is the diluent of the TGFRI pharmacological inhibitor SD208 and serves as control medium. The ability of NRP2 to promote the formation of Colo320 colonies in soft agar cultures was not observed in the presence of TGFRI pharmacological inhibition, in presence or not of TGF-β1. 10ng/mL TGF-β1 treatment increased the number of colonies of Colo320^siRNA-ctrl^, whereas this same treatment has no impact on Colo320^siRNA-NRP2^ cells. Data represent means of triplicates plus or minus the standard error (SE). The presence of TGFRI pharmacological inhibitor decreased the ability of Colo320 to form colony in soft agar cultures.
(TIF)

Table S1 **Review of the publications mentioning the expression of NRP2 in cancer samples (x indicates the identification of NRP expression, while ND (note done) indicates the absence of investigation related to NRP1 or 2 expression in tumor cell lines.**
(DOCX)

Table S2 **NRP2 expression in tumor cell lines.**
(DOCX)
