# Supplementary figures and images for: Neuropilin-2 Expression Promotes TGF-β1-Mediated Epithelial to Mesenchymal Transition in Colorectal Cancer Cells
Source: PLoS One. 2011 Jul 1;6(7):e20444. doi: 10.1371/journal.pone.0020444 (PMC3128581; doi:10.1371/journal.pone.0020444)

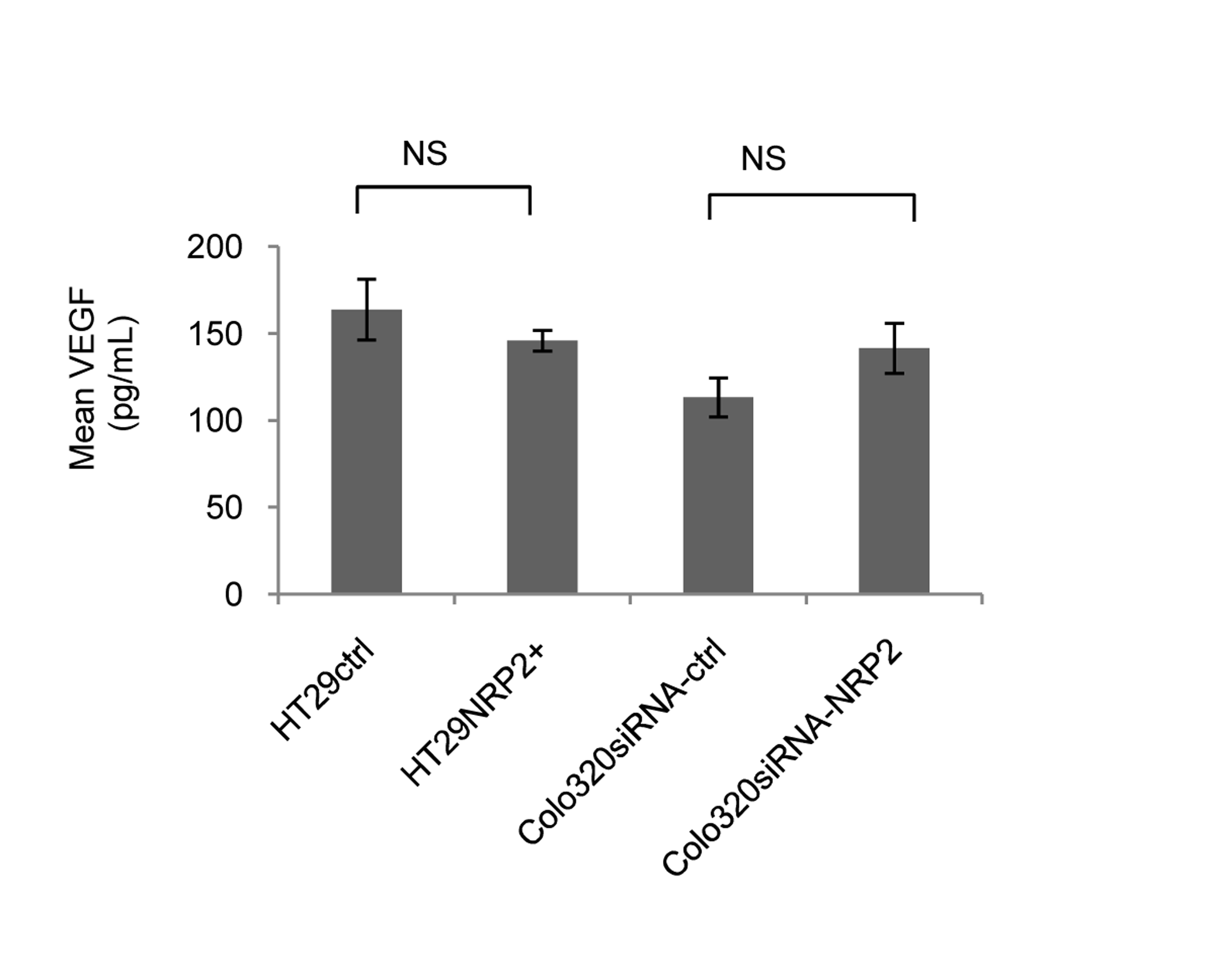

Supplement: Figure S1 — VEGF-A production was monitored by ELISA assay in HT29ctrl, HT29NRP2, Colo320siRNA-NRP2 and Colo320siRNA-ctrl cells. Low level of VEGF-A was detected and no significative difference in secretion of VEGF-A was noticed in all cell lines analyzed. NRP2 expression did not influence VEGF production. (TIF) [file pone.0020444.s001.tif]

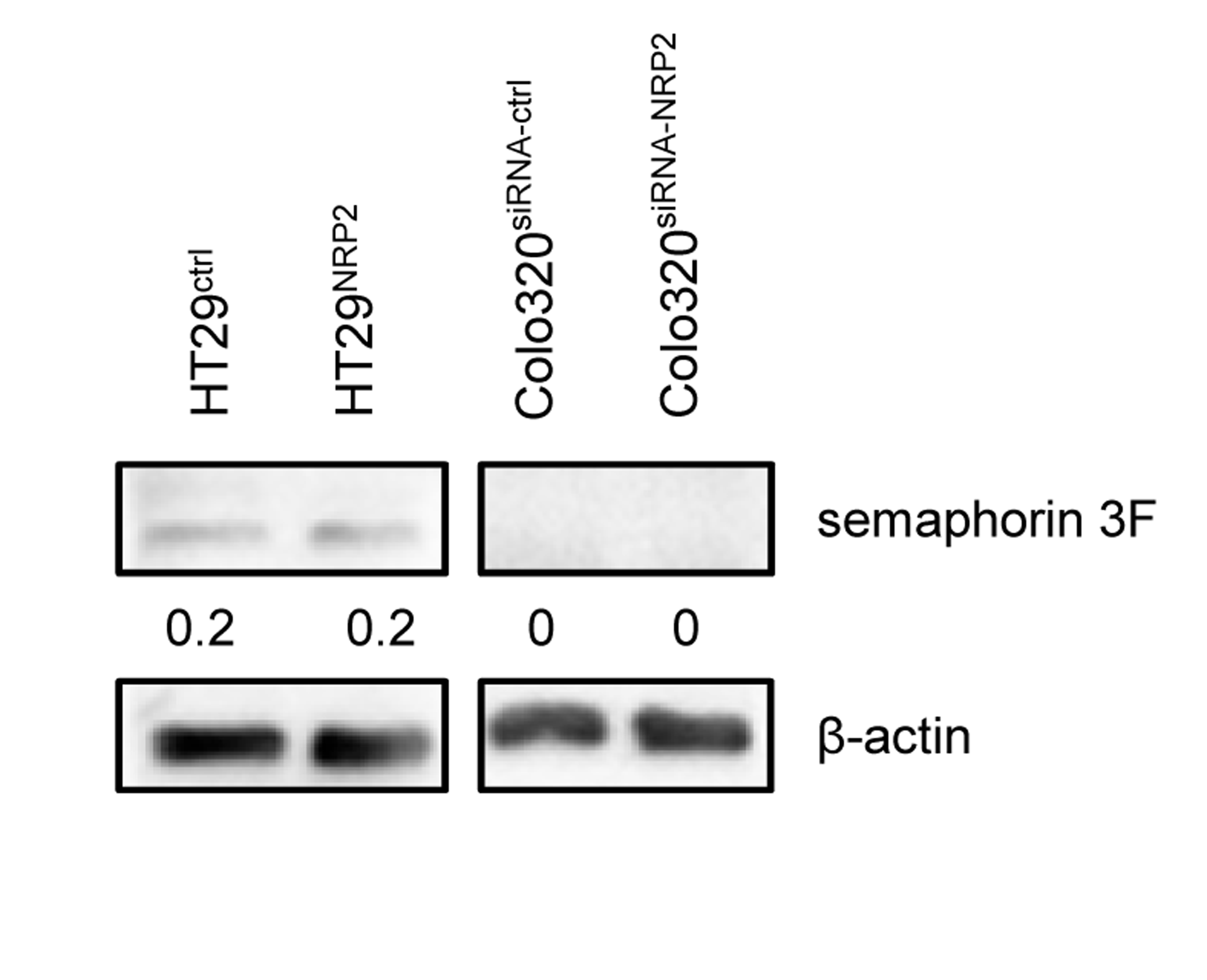

Supplement: Figure S2 — Western blotting experiments showed that HT29ctrl and HT29NRP2 express the same level of semaphorin 3F, whereas no semaphorin 3F was found in Colo320 cells. β-actin was used as a control of protein loading. (TIF) [file pone.0020444.s002.tif]

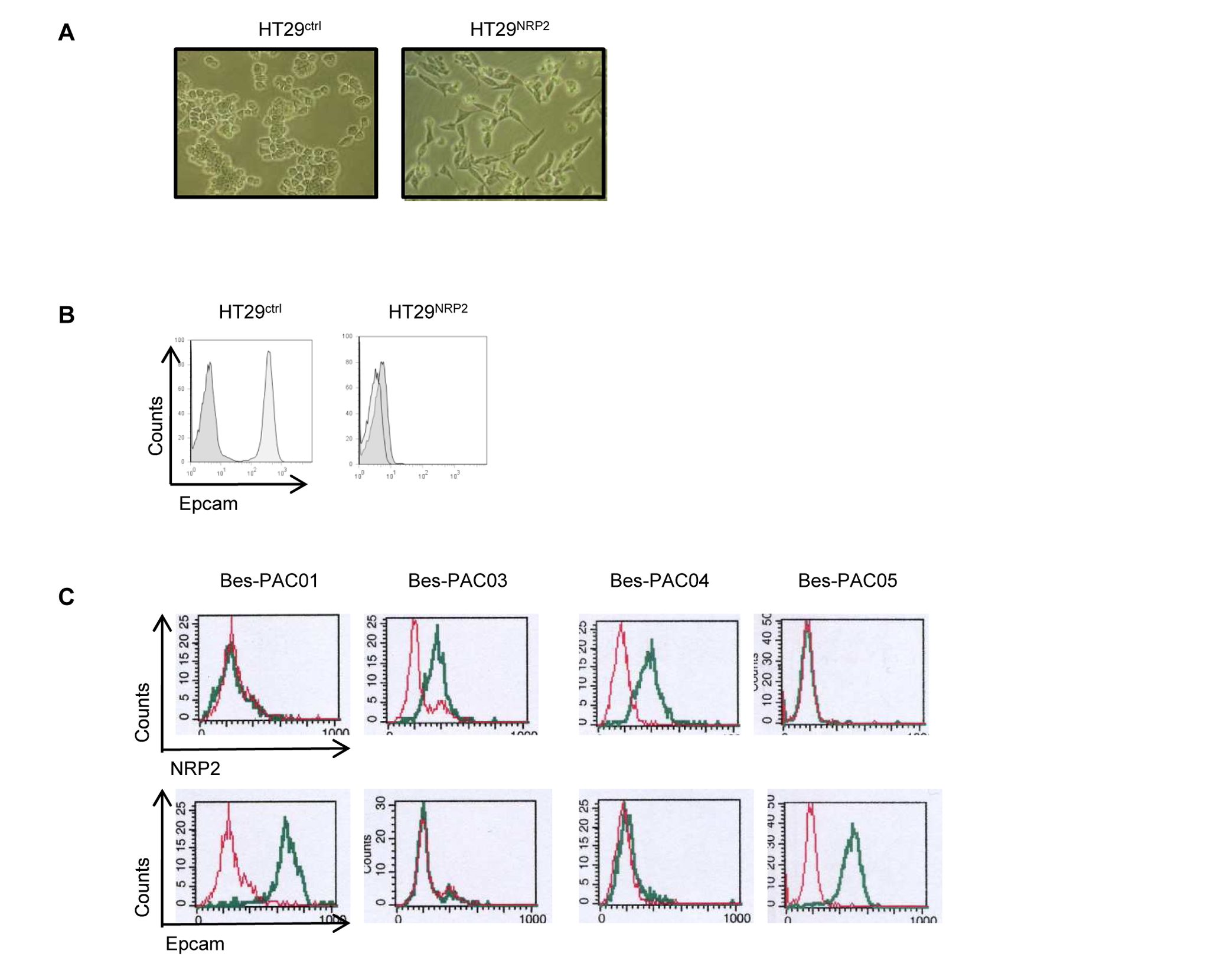

Supplement: Figure S3 — A, HT29ctrl and HT29NRP2 were cultured and observed using a light microscope. Cells were photographed at magnification *40. HT29NRP2 displayed a fibroblastic-like shape whereas HT29ctrl exhibited a cuboidal phenotype. B, FACS analysis reveals that NRP2 expression induces decrease of Epcam protein in HT29NRP2 cells. C, FACS analysis of NRP2 and Epcam proteins in pancreatic cancer cell lines. NRP2 and Epcam are inversely correlated. (red: isotype, green: anti-NRP2 or anti-Epcam antibodies). (TIF) [file pone.0020444.s003.tif]

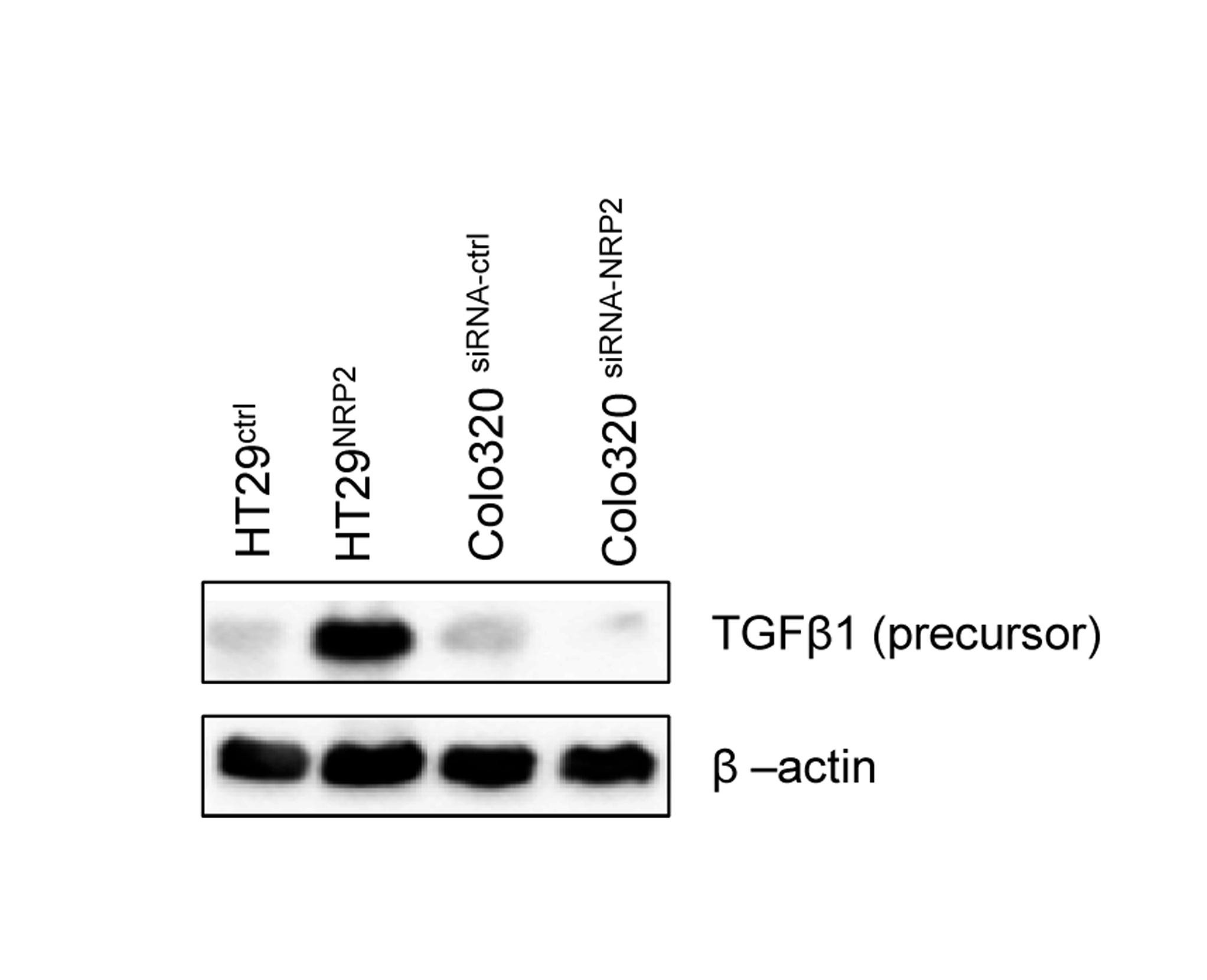

Supplement: Figure S4 — Western-blotting experiments shows that NRP2 positive cells (Colo320siRNA-ctrl and HT29NRP2) secreted higher level of TGFβ1 precursors than NRP2 negative cells (HT29ctrl and Colo320siRNA-NRP2). β-actin was used as a control of protein loading. (TIF) [file pone.0020444.s004.tif]

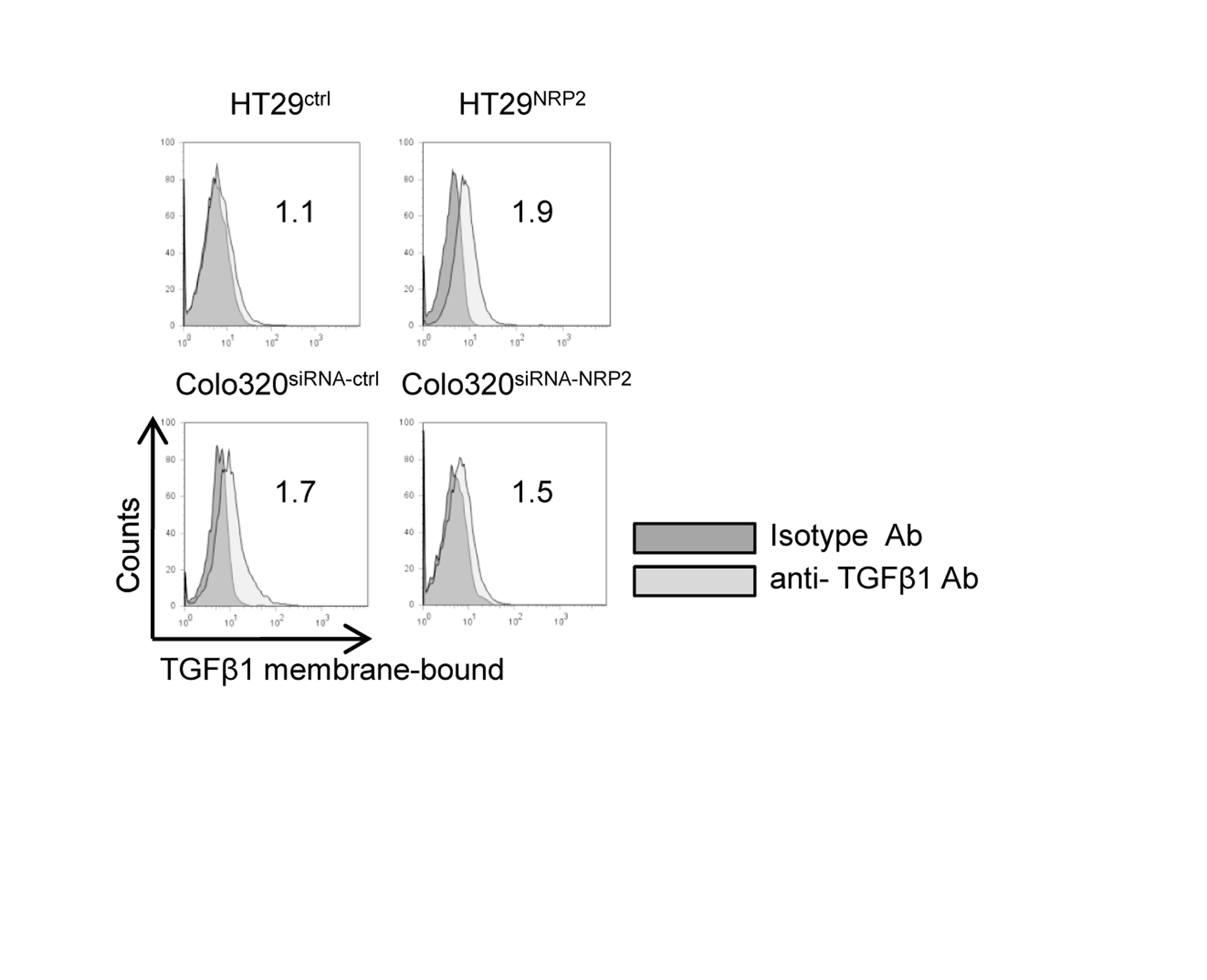

Supplement: Figure S5 — The level of membrane associated-TGF-β1 was assessed by FACS analysis in HT29ctrl, HT29NRP2, Colo320siRNA-NRP2 and Colo320siRNA-ctrl. Relative Fluorescence Intensity (RFI) was calculated. NRP2 expression is associated with an increase of TGF-β1 on HT29 cytoplasmic membrane, while treatment of Colo320 with NRP2-siRNA decreased membrane-bound TGF-β1. (TIF) [file pone.0020444.s005.tif]

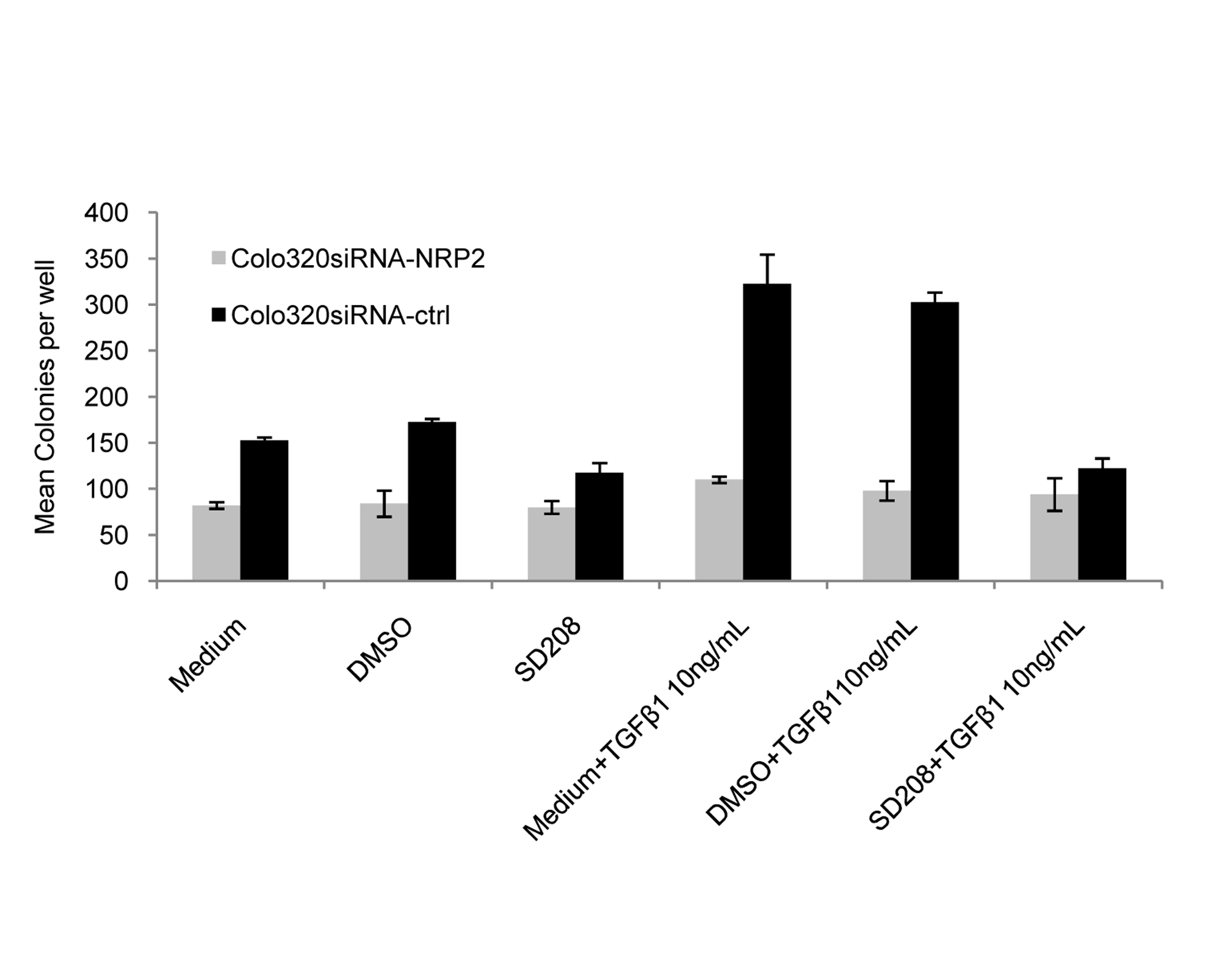

Supplement: Figure S6 — Colo320siRNA-ctrl or Colo320siRNA-NRP2 were cultured in soft-agar containing medium for 10 days with or without TGFRI pharmacological inhibitor (SD208, 10µMol). DMSO is the diluent of the TGFRI pharmacological inhibitor SD208 and serves as control medium. The ability of NRP2 to promote the formation of Colo320 colonies in soft agar cultures was not observed in the presence of TGFRI pharmacological inhibition, in presence or not of TGF-β1. 10ng/mL TGF-β1 treatment increased the number of colonies of Colo320siRNA-ctrl, whereas this same treatment has no impact on Colo320siRNA-NRP2 cells. Data represent means of triplicates plus or minus the standard error (SE). The presence of TGFRI pharmacological inhibitor decreased the ability of Colo320 to form colony in soft agar cultures. (TIF) [file pone.0020444.s006.tif]
