# Supplementary material for: Neuropilin-2 Expression Promotes TGF-β1-Mediated Epithelial to Mesenchymal Transition in Colorectal Cancer Cells
Source: PLoS One. 2011 Jul 1;6(7):e20444. doi: 10.1371/journal.pone.0020444 (PMC3128581; doi:10.1371/journal.pone.0020444)
Supplement: Table S1 — Review of the publications mentioning the expression of NRP2 in cancer samples (x indicates the identification of NRP expression, while ND (note done) indicates the absence of investigation related to NRP1 or 2 expression in tumor cell lines. (DOCX) [file pone.0020444.s007.docx]

| **Tumors** | **NRP1** | **NRP2** | **References** |
| --- | --- | --- | --- |
| **Brain tumors** | | | |
| Astrocytomas | x | ND | Ding Het al. (2000), Int J Cancer 88: 584-592. |
| Neuroblastomas | x | x | Fakhari M et al. (2002),Cancer 94: 258-263. |
| Gliomas | x | x | Rieger J et al. (2003), Glia 42: 379-389. |
|  | x | ND | Osada R et al. (2006), Hum Pathol 37: 1414-1425. |
| Glioblastomas | x | ND | Broholm H et al. (2004), APMIS 112: 257-263. |
| Pituitary tumors | x | ND | Onofri C et al. (2006), J Endocrinol 191. |
| **Digestive tumors** | | | |
| Endocrine pancreatic tumors | ND | x | Cohen T et al. (2002), J Pathol 198: 77-82. |
| Pancreatic adenocarcinomas | x | ND | Parikh AA et al. (2003), Cancer 98: 720-729. |
|  | x | x | Fukahi K et al. (2004), Clin Cancer Res 10: 581-590. |
|  | x | x | Li M et al. (2004), Cancer 101: 2341-2350. |
|  | x | ND | Feurino LW et al. (2007), Cancer Biol Ther 6: 1096-1100. |
|  | x | x | Dallas NA et al. (2008), Clin Cancer Res 14: 8052-8060. |
| Gastric cancer | x | ND | Akagi M et al. (2003), Br J Cancer 88: 796-802. |
|  | x | ND | Hansel DE et al. (2004), Am J Surg Pathol 28: 347-356. |
| Colon cancer | x | ND | Parikh AA et al. (2004), Am J Path 164: 2139-2151. |
|  | x | ND | Ochiumi T et al. (2006), Int J Oncol 29: 105-116. |
|  | ND | x | Gray MJ et al. (2008), J Natl Cancer Inst 100 109 - 120. |
| **Leukemias** | | | |
| Acute Myeloid Leukemia (AML) | x | ND | Kreuter M et al. (2006), Leukemia 20: 1950-1954. |
|  | x | ND | Kreuter M et al. (2007), Eur J Haematol 79: 392-397. |
|  | x | x | Vales A et al. (2007), Leuk Lymphoma 48: 1997-2007. |
|  | x | ND | Lu L et al. (2008), Leuk Lymphoma 49: 331-338. |
| Chronic lymphocytic leukemia B | x | ND | Nowakowski GS et al. (2008), Leuk Res 32: 1634-1636. |
| **Other solid tumors** | | | |
| Breast cancers | x | ND | Stephenson JM et al. (2002), Int J Cancer 101: 409-414. |
|  | x | ND | Ghosh S et al. ( 2008), Hum Pathol 39: 1835-1843. |
| NSCLC | x | x | Kawakami T et al. (2002), Cancer 95: 2196-2201. |
|  | x | x | Lantuéjoul S et al. (2003), J Pathol 200: 336-347. |
| Lung cancers | x | x | Tomizawa Y et al. (2001), Proc Natl Acad Sci U S A 98: 13954-13959. |
| Melanomas | x | x | Lacal PM et al. (2000), J Invest Dermatol 115: 1000-1007. |
|  | x | ND | Straume O et al. (2003), Angiogenesis 6: 295-301. |
| Prostate cancers | x | ND | Latil A et al. (2000), Int J Cancer 89: 167-171. |
|  | x | ND | Vanveldhuizen PJ et al. (2003), Oncol Rep 10: 1067-1071. |
| Laryngeal carcinomas and papillomas | x | ND | Zhang S et al. (2006), Lin Chuang Er Bi Yan Hou Ke Za Zhi 20: 634-635. |
| Salivary adenoid cystic carcinoma | ND | x | Cai Y et al. (2010), Pathol Res Pract. |
| Infantile hemangiomas | ND | x | Calicchio ML et al. (2009), Am J Pathol 174: 1638-1649. |
| Ovarian carcinomas | x | ND | Hall GH et al. (2005), Int J Oncol 27: 1283-1288. |
|  | x | x | Osada R et al. (2006), Hum Pathol 37: 1414-1425. |
|  | x | ND | Baba T et al. (2007), Gynecol Oncol 105: 703-711. |
| Bladder cancers | ND | x | Sanchez-Carbayo M et al. (2003), Am J Pathol 163: 505-516. |
| Osteosarcomas | ND | x | Handa A et al. (2000), Int J Oncol 17: 291-295. |

**Supplementary table 1. Review of the publications mentioning the expression of NRP2 in cancer samples (x indicates the identification of NRP expression, while ND (note done) indicates the absence of investigation related to NRP1 or 2 expression in tumor cell lines.**
