# Supplementary material for: Neuropilin-2 Expression Promotes TGF-β1-Mediated Epithelial to Mesenchymal Transition in Colorectal Cancer Cells
Source: PLoS One. 2011 Jul 1;6(7):e20444. doi: 10.1371/journal.pone.0020444 (PMC3128581; doi:10.1371/journal.pone.0020444)
Supplement: Table S2 — NRP2 expression in tumor cell lines. (DOCX) [file pone.0020444.s008.docx]

| **HT29ctrl/HT29NRP2** | **Significativity at T24** | **Significativity at T48** | **Significativity at T72** |
| --- | --- | --- | --- |
| **Experiment n°1** | Yes | Yes | Yes |
| **Experiment n°2** | Yes | Yes | Yes |
| **Experiment n°3** | No | No | Yes |

| **Colo320siRNA-ctrl/Colo320siRNA-nrp2** | **Significativity at T24** | **Significativity at T48** | **Significativity at T72** |
| --- | --- | --- | --- |
| **Experiment n°1** | Yes | Yes | Yes |
| **Experiment n°2** | No | No | Yes |
| **Experiment n°3** | No | Yes | Yes |

**Supplementary Table 2. NRP2 expression in tumor cell lines.**

3 MTT experiments with each type of colorectal cancer cells (HT29^ctrl^, HT29^NRP2^, Colo320^siRNA-ctrl^ and Colo320^siRNA-NRP2^) have been realized to confirm NRP2 role in cell proliferation. Cell proliferation in vitro was analyzed with the tetrazolium salt 3-(4,5-dimethylthiazol-2-yl)-2,5 diphenyltetrazolium bromide (MTT). Briefly, 4000 cells per well were seeded in 96-well micro-plates containing 100 µL of medium per well. These experiments and their degree of significativity are reported in the following table: (significativity: Student Test, p<0.05) after 24, 48 and 72 hours of culture for each experiment (T24, T48, T72).
